# Externally Validated Probabilistic Modeling of a Predefined Entecavir Resistance Pathway in HBV Using Independent Public Repositories

**DOI:** 10.3390/v18060610

**Published:** 2026-05-27

**Authors:** Christelos Kapatais, Fanie Karaoulani, Sotirios P. Fortis, Matina Saritzoglou, Nikolaos Martsoukos, Andreas Kapatais

**Affiliations:** 1 1st Department of Respiratory Medicine, Sotiria Hospital, Medical School, National and Kapodistrian University of Athens, 11527 Athens, Greece; 2 Biopathological-Biochemical Department, Psychiatric Hospital of Attica “Dromokaiteio”, 12461 Athens, Greece; faniekaraoulani@yahoo.gr; 3 Laboratory of Reliability and Quality Control in Laboratory Hematology (HemQcR), Department of Biomedical Sciences, School of Health & Caring Sciences, University of West Attica (UniWA), Ag. Spyridonos Street, 12243 Athens, Greece; sfortis@uniwa.gr (S.P.F.); nickmartsoukos@gmail.com (N.M.); 4 3rd Department of Pathology, Sotiria Hospital, Medical School, National and Kapodistrian University of Athens, 11527 Athens, Greece; matinasartz@gmail.com; 5 1st Department of Internal Medicine, General Hospital of Nikea “Agios Panteleimon”, 18454 Piraeus, Greece; 6 1st Department of Internal Medicine, General Hospital of West Attica “Agia Varvara”, 12351 Athens, Greece; andreaskapatais@yahoo.gr

**Keywords:** hepatitis B virus, entecavir, drug resistance, machine learning, external validation, viral genomics

## Abstract

Background: Accurate interpretation of hepatitis B virus (HBV) polymerase sequences is essential for identifying antiviral resistance, particularly for high-genetic-barrier agents such as entecavir. Current resistance interpretation relies largely on deterministic rule-based systems that do not quantify uncertainty and are difficult to evaluate across independent datasets. We aimed to develop and externally validate a transparent probabilistic framework for reconstructing a predefined entecavir resistance pathway from HBV polymerase sequences. Methods: HBV polymerase sequences were retrieved from the NCBI GenBank database and curated through translation, quality control, and deduplication to create the development dataset. Reverse transcriptase (RT) positions were indexed using motif-anchored numbering based on the YMDD-family motif. A genotypic proxy for the entecavir resistance pathway was defined by lamivudine-associated background substitutions combined with entecavir-associated RT substitutions. A logistic regression model with probability calibration was trained and internally validated using prespecified performance metrics and thresholds. External validation was performed on an independent HBVdb dataset with preprocessing, model parameters, and thresholds frozen prior to evaluation. Results: The development dataset comprised 1174 unique polymerase sequences, of which 268 met the resistance pathway definition. Internal validation demonstrated perfect discrimination, consistent with the deterministic genotypic definition of the outcome. External validation on 11,513 independent HBVdb sequences demonstrated reproducible performance across repositories despite a markedly lower prevalence of the resistance pathway (2.2%), with preserved discrimination and stable threshold-based performance. Conclusions: This study presents a transparent and externally validated machine learning framework for probabilistic identification of the entecavir resistance pathway in HBV. The approach provides a transparent and reproducible probabilistic formalization of an established genotypic resistance definition and may serve as a methodological framework for standardized sequence-based resistance interpretation.

## 1. Introduction

Chronic hepatitis B virus (HBV) infection remains a major global health burden, with an estimated 296 million people affected worldwide and a substantial proportion at risk of progressive liver disease, including cirrhosis and hepatocellular carcinoma [1]. Long-term suppression of viral replication using nucleos(t)ide analogues represents the cornerstone of antiviral therapy, with high-barrier agents such as entecavir and tenofovir forming the backbone of current treatment strategies [2,3]. Despite their favorable resistance profiles, antiviral resistance continues to be a clinically relevant concern, particularly in treatment-experienced patients and in settings where sequential therapy or suboptimal adherence occurs [4].

Genotypic resistance to HBV nucleos(t)ide analogues arises from specific amino-acid substitutions within the viral polymerase reverse transcriptase (RT) domain. For entecavir, resistance is typically observed in the context of a lamivudine-associated resistance background, followed by the accumulation of additional substitutions at defined RT positions [5,6,7]. Interpretation of these resistance pathways has traditionally relied on rule-based algorithms and expert-curated mutation lists. While such approaches have been instrumental in clinical practice, they generally provide deterministic binary classifications and may be difficult to evaluate quantitatively across heterogeneous sequence datasets [8,9,10,11,12,13,14]. In contrast, probabilistic prediction frameworks can provide calibrated risk estimates, support threshold-based interpretation, and enable formal assessment of discrimination, calibration, and transportability across independent datasets [11,12,13,14].

The increasing availability of publicly accessible viral sequence repositories, such as NCBI GenBank and HBVdb, offers an opportunity to re-examine resistance interpretation using data-driven methods. However, the application of machine learning to viral genotypic resistance has been limited by concerns regarding transparency, reproducibility, overfitting, and lack of external validation, issues that have been extensively documented across biomedical prediction models [10,11,12,13,14,15,16]. In particular, many published models rely on complex architectures that obscure biological interpretation or fail to evaluate calibration and clinical utility, limiting their translational relevance.

In this context, there is a need for computational frameworks that balance interpretability with statistical rigor and that explicitly demonstrate generalizability across independent datasets. Rather than aiming to predict clinical outcomes, such frameworks can serve as probabilistic tools for interpreting established genotypic resistance pathways, complementing existing rule-based systems while preserving transparency. Calibration, threshold selection, and decision-analytic evaluation are essential components of this process, yet they remain underreported in the viral hepatitis literature [13,14,17]. The objective of the present study was therefore not de novo discovery of resistance determinants, but development of a transparent probabilistic framework for standardized interpretation and external validation of predefined resistance-associated sequence patterns.

In the present study, we developed and externally validated a transparent, probabilistic model for identifying the entecavir resistance pathway based on HBV polymerase RT sequence features derived from publicly available data. Using a rigorously prespecified pipeline, we trained a calibrated logistic regression model on a curated NCBI-derived dataset and evaluated its performance on an independent HBVdb cohort under conditions of marked prevalence shift. Emphasis was placed on reproducibility, avoidance of information leakage, and comprehensive reporting in accordance with TRIPOD-AI recommendations [18]. This work aims to provide a reference framework for sequence-based resistance pathway interpretation rather than a replacement for clinical decision-making.

## 2. Materials and Methods

### 2.1. Study Design and Data Sources

This study was designed as a computational analysis of publicly available HBV polymerase sequences aimed at developing and externally validating a probabilistic model for identification of the entecavir resistance pathway. The analytical workflow was prespecified prior to model fitting and included data acquisition, sequence curation, feature extraction, model development, internal validation, and independent external validation.

HBV polymerase coding sequences used for model development were obtained from the NCBI Nucleotide database (National Center for Biotechnology Information, Bethesda, MD, USA) [15]. An independent external validation cohort was derived from HBVdb (Institut National de la Santé et de la Recherche Médicale, Paris, France), a curated hepatitis virus sequence database developed to support virological and resistance research [9]. These data sources were selected because they provide large-scale, publicly accessible viral sequence data and differ in curation practices and submission pipelines, allowing evaluation of model generalizability across distinct repositories. Details of sequence retrieval, translation, quality control, and deduplication are provided in Supplementary Sections S2 and S6.

Following sequence retrieval, translation, quality control, and deduplication, the final development dataset comprised 1174 unique HBV polymerase amino-acid sequences, each representing an independent viral sequence after removal of duplicates and low-quality records. Among these, 268 sequences met the predefined genotypic criteria for the entecavir resistance pathway, while the remaining sequences were classified as negative for the pathway proxy. For internal validation, the development dataset was partitioned into a training set (*n* = 976) and an internal validation set (*n* = 198) using a stratified split to preserve outcome prevalence.

**Figure 1:**
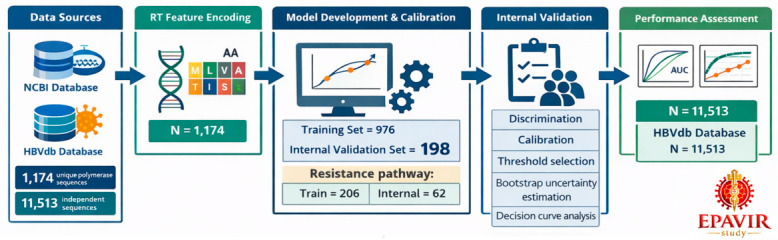
Overview of the study workflow for model development and validation. Schematic representation of the prespecified analytical workflow used for model development and validation. HBV polymerase coding sequences were retrieved from publicly available databases and subjected to translation, quality control, and deduplication. RT amino-acid features were extracted using motif-anchored RT numbering, and a predefined panel of RT positions was encoded for model input. A logistic regression model was trained on the curated NCBI-derived development cohort, followed by internal validation, probability calibration, and threshold selection. External validation was performed using an independent HBVdb derived cohort, with the trained model, feature encoder, calibration parameters, and decision thresholds applied unchanged. Performance was assessed using discrimination, calibration, and decision-analytic metrics. Abbreviations: NCBI = National Center for Biotechnology Information; HBVdb = Hepatitis B Virus Database; RT = reverse transcriptase; AA = amino acid; AUC = Area Under Curve.

External validation was conducted on an independent cohort of 11,513 HBV polymerase sequences obtained from HBVdb, which were processed using the same translation, quality control, and feature extraction procedures but were not used at any stage of model development or calibration. These sequence counts are summarized graphically in Figure 1 to provide a high-level overview of dataset flow across development, internal validation, and external validation stages.

### 2.2. Feature Extraction and Outcome Definition

A predefined panel of reverse transcriptase (RT) positions was selected a prioribased on established associations with nucleos(t)ide analogue resistance and local sequence context, as described in international clinical and virological guidelines [3,4]. The panel included RT positions 80, 84, 91, 169, 173, 180, 181, 184, 191, 194, 200, 202, 204, 215, 233, 236, 250, 256, and 269, which encompass key residues implicated in lamivudine- and entecavir-associated resistance. Amino-acid residues at each selected position were treated as categorical variables and encoded using one-hot encoding. RT positions were intentionally restricted to previously reported resistance-associated regions to preserve biological interpretability and consistency with established HBV resistance literature [4,8,9]. Alternative data-driven feature selection strategies were not explored because the aim of the study was validation of a predefined resistance pathway rather than de novo mutation discovery. Feature extraction and encoding procedures are described in Supplementary Section S3, and the complete RT feature panel and amino-acid distributions are reported in Supplementary Table S1.

The outcome variable was defined as a genotypic proxy for the entecavir resistance pathway. Sequences were labeled as positive if they exhibited a lamivudine-associated resistance background together with at least one substitution at RT positions previously associated with entecavir resistance. This definition reflects a well-characterized resistance pathway rather than a clinical endpoint and is supported by longitudinal and mechanistic studies of HBV antiviral resistance [4,5,6,7]. The same outcome definition was applied uniformly across the development and external validation datasets. Further details are provided in Supplementary Section S2 and Table S2.

### 2.3. Model Development and Internal Validation

A logistic regression model was selected a priori to balance interpretability and statistical rigor, consistent with recommendations for transparent prediction modeling in biomedical research [11,12]. Model training and internal validation were performed using a stratified train (*n* = 976)/test (*n* = 198) split of the development dataset. Class imbalance was addressed through the use of class weighting during model fitting. Probability calibration was applied to support threshold-based interpretation of model outputs.

Internal validation procedures included assessment of model discrimination, calibration, and threshold-based performance. Discrimination was evaluated using the area under the receiver operating characteristic and precision–recall curves, while calibration was assessed using calibration plots and the Brier score [13]. Threshold-based performance was summarized using sensitivity, specificity, positive predictive value (PPV), negative predictive value (NPV), precision, accuracy, F1 score, and Matthews correlation coefficient, with decision thresholds selected using prespecified discrimination-oriented criteria, including maximization of the Youden index and F1 score. Threshold performance was subsequently evaluated using confusion matrices, calibration analyses, and threshold-dependent classification metrics across internal and external validation datasets. Uncertainty around performance estimates was quantified using nonparametric bootstrap resampling. Full details of model specification, calibration procedures, threshold selection, and internal validation analyses are provided in Supplementary Materials Sections S4 and S5.

### 2.4. External Validation

External validation was conducted using an independently curated HBVdb-derived cohort distinct from the NCBI-derived development dataset. External sequences underwent separate preprocessing, including six-frame translation, quality-control filtering, motif screening, and deduplication using sequence hash matching to minimize overlap with the development cohort. Preparation of the external dataset was performed independently of model development. The trained model, feature encoder, calibration parameters, and decision thresholds were frozen prior to external evaluation and applied unchanged to the validation dataset. This design was intended to assess model transportability across heterogeneous public sequence repositories processed through partially independent curation workflows.

External model performance was assessed using the same discrimination, calibration, and threshold-based metrics applied during internal validation, including ROC curve and precision–recall analyses, calibration assessment using calibration plots and the Brier score, and threshold-based performance measures. Decision curve analysis was performed as an exploratory assessment of threshold-dependent classification behavior within the predefined genotypic framework [13]. No retraining, recalibration, or threshold optimization was performed during external validation. Full details of external validation analyses are provided in Supplementary Materials Section S6 and S7.

### 2.5. Performance Assessment and Reporting Standards

The study was conducted and reported in accordance with contemporary recommendations for transparent reporting of prediction model development and validation, including guidance for artificial intelligence-based models [12,16,18]. Detailed descriptions of performance metrics, threshold selection, calibration, and decision-analytic evaluation are provided in the relevant internal and external validation sections and in the Supplementary Materials. All scripts used for data processing, model development, and evaluation are provided in the Supplementary Materials to facilitate full reproducibility. All analyses were performed in Python version 3.11.9 (Python Software Foundation, Wilmington, DE, USA) using scikit-learn (INRIA, Paris, France), pandas (NumFOCUS, Austin, TX, USA), NumPy (NumPy Developers, Austin, TX, USA), Matplotlib 3.10.0 (Matplotlib Development Team, Austin, TX, USA), Biopython (Biopython Project), and joblib (Joblib Developers).

### 2.6. Ethics

This study analyzed publicly available, non-identifiable viral sequence data from public repositories. Ethics approval and consent to participate were therefore not required.

## 3. Results

### 3.1. Development Cohort Characteristics

After sequence retrieval, translation, quality control, and deduplication, the development dataset comprised 1174 unique HBV polymerase amino-acid sequences, each representing an independent viral sequence. Of these, 268 sequences (22.8%) fulfilled the predefined genotypic criteria for the entecavir resistance pathway proxy, while the remaining sequences were classified as negative. The distribution of reverse transcriptase amino-acid features and resistance pathway components in the development cohort is summarized in Supplementary Materials Tables S1 and S2. The development dataset was partitioned into training (*n* = 976) and internal validation (*n* = 198) subsets using stratified sampling to preserve outcome prevalence. No sequence overlap was observed between the development and external validation cohorts.

### 3.2. Internal Validation Performance

**Figure 2:**
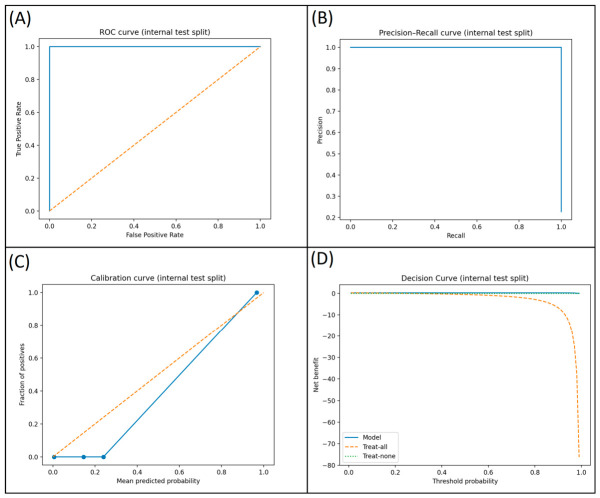
Internal validation performance of the calibrated model. This figure illustrates the comprehensive internal validation diagnostics for the calibrated logistic regression model evaluated on the held-out development test set. (**A**) shows the receiver operating characteristic (ROC) curve, illustrating discrimination across all probability thresholds. (**B**) presents the precision–recall (PR) curve, which complements the ROC analysis by highlighting performance under outcome imbalance. (**C**) displays the calibration curve, depicting the agreement between predicted probabilities and observed frequencies of the entecavir resistance pathway proxy; the diagonal line represents perfect calibration. (**D**) shows decision curve analysis, summarizing the decision-analytic behavior of the model across a range of decision thresholds compared with default “treat-all” and “treat-none” strategies. All analyses were performed using the frozen model, feature encoder, calibration parameters, and prespecified decision thresholds established during internal validation. No retraining or recalibration was conducted at this stage.

During internal validation, the model demonstrated perfect discrimination between sequences classified as positive and negative for the entecavir resistance pathway proxy, with area under the receiver operating characteristic and precision–recall curves equal to 1.00 (Figure 2A,B). Calibration analysis showed close agreement between predicted probabilities and observed outcome frequencies across the full range of predictions, reflected by a low Brier score and near-ideal calibration curves (Figure 2C).

Threshold-based performance assessment using prespecified decision thresholds yielded perfect sensitivity, specificity, precision, and accuracy in the internal validation set. Threshold-specific confusion matrices for internal validation are shown in Supplementary Figure S3. Bootstrap resampling confirmed stability of these performance estimates, with narrow confidence intervals across all evaluated metrics. Decision curve analysis demonstrated stable threshold-dependent classification behavior across a range of probability thresholds (Figure 2D). Direct comparison with a deterministic rule-based classifier using the same predefined resistance mutation criteria demonstrated near-equivalent classification performance and complete concordance in internal validation, consistent with the deterministic construction of the endpoint definition (Supplementary Table S3). The identical threshold identified by both the Youden index and F1 optimization procedures supported selection of a stable operating threshold balancing sensitivity, specificity, and precision within the internally validated dataset.

The objective of the present study was therefore not de novo discovery of resistance determinants, but development of a transparent probabilistic framework for standardized interpretation and external validation of predefined resistance-associated sequence patterns. Given the deterministic relationship between the predefined RT features and the outcome definition, these results reflect the model’s ability to reproduce a predefined genotypic resistance pathway rather than predict an independent clinical outcome. The primary purpose of the framework was therefore not to outperform existing rule-based interpretation systems in reconstructing known resistance definitions, but to provide a transparent probabilistic implementation that enables calibration assessment, threshold-based evaluation, and formal external validation across independent datasets.

### 3.3. External Validation and Generalizability

External validation was performed using an independent cohort of 11,513 HBV polymerase sequences derived from HBVdb, processed using the same quality control and feature extraction procedures but not used at any stage of model development. Among these sequences, 248 (2.2%) met the predefined criteria for the entecavir resistance pathway proxy, reflecting a marked prevalence shift relative to the development cohort.

**Figure 3:**
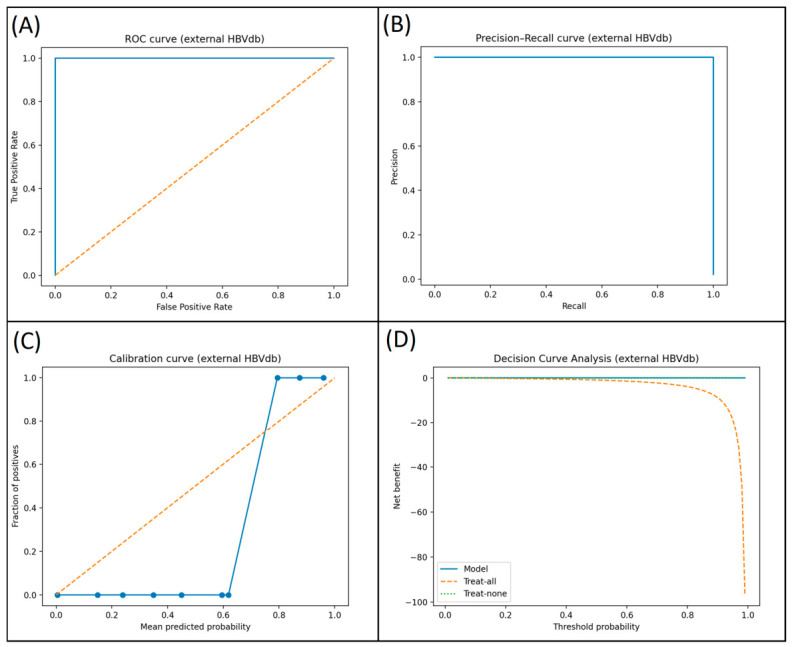
External validation performance of the calibrated model evaluated on an independent HBVdb derived cohort. (**A**) shows the ROC curve, summarizing the model’s ability to discriminate between sequences positive and negative for the entecavir resistance pathway proxy across all probability thresholds. (**B**) presents the PR curve, highlighting model performance under conditions of marked outcome imbalance in the external dataset. (**C**) displays the calibration curve, illustrating the agreement between predicted probabilities and observed frequencies of the resistance pathway in the external cohort; the diagonal line represents ideal calibration. (**D**) shows decision curve analysis, depicting the decision-analytic behavior of model-guided classification across a range of threshold probabilities compared with default “treat-all” and “treat-none” strategies. All analyses were performed using the fully trained and calibrated model with frozen feature encoder, calibration parameters, and decision thresholds derived during internal validation. No retraining, recalibration, or threshold optimization was performed prior to external evaluation. Abbreviations: ROC = receiver operating characteristic; HBVdb = Hepatitis B Virus Database.

Despite this shift, the model maintained excellent discriminatory performance in external validation, with high area under the receiver operating characteristic and precision–recall curves (Figure 3A,B). Calibration analysis demonstrated preservation of probabilistic accuracy, with predicted risks remaining well aligned with observed outcome frequencies (Figure 3C). Threshold-based performance metrics remained robust when applying the frozen decision thresholds derived during internal validation. Despite the low prevalence of the predefined resistance pathway in the external cohort, negative predictive value remained high across evaluated thresholds, while positive predictive value varied according to the selected operating threshold (Supplementary Table S3).

Decision curve analysis in the external cohort indicated sustained net benefit of model-based classification across clinically relevant threshold probabilities compared with default strategies (Figure 3D), supporting reproducibility of probabilistic classification behavior across repositories with differing pathway prevalence.

### 3.4. Summary of Model Performance

Across internal and external validation, the model consistently demonstrated high discrimination, good calibration, and stable threshold-based performance under conditions of dataset heterogeneity and prevalence shift. These findings support the feasibility of using a transparent, probabilistic framework to identify established HBV genotypic resistance pathways from polymerase sequence data while preserving reproducibility and external generalizability.

## 4. Discussion

In this study, we developed and externally validated a transparent probabilistic framework for identifying an established entecavir resistance pathway using HBV polymerase reverse transcriptase sequences from independent public repositories. The main finding is that a biologically constrained and prespecified model can reproduce a known resistance pathway with high internal consistency while maintaining performance in an external dataset with substantially different pathway prevalence.

These results are consistent with the established understanding of entecavir resistance as a stepwise process that usually develops on a lamivudine-resistant background. Earlier mechanistic and clinical studies showed that substitutions such as rtL180M and rtM204V/I provide the background on which additional changes at rt184, rt202, or rt250 can reduce susceptibility to entecavir [4,5,6,7,19,20,21]. Therefore, the present model should not be interpreted as discovering a new resistance mechanism. Rather, it probabilistically formalizes and externally validates an already recognized resistance pathway within a transparent and reproducible analytical framework. Given that the outcome definition was directly derived from predefined resistance-associated substitutions, a deterministic rule-based classifier based on the same mutation criteria would be expected to produce near-equivalent classification results by construction. This distinction is important because the model’s value lies not in replacing biological knowledge, but in converting that knowledge into a transparent, testable, and externally validated analytical pipeline.

The near-perfect internal performance observed in this study was expected given the deterministic relationship between the predefined RT features and the outcome definition. Similar concerns have been raised in prediction modeling more broadly, where very high internal performance may reflect outcome construction, feature leakage, or insufficient separation between development and evaluation data [11,12,13,18]. In our study, this risk was addressed by prespecifying the feature panel, freezing the encoder and model parameters, and performing external validation in an independent HBVdb-derived cohort after cross-database deduplication. The preservation of performance under a marked prevalence shift supports the robustness of the motif-anchored feature representation and reduces the likelihood that the model simply captured repository-specific artifacts.

A methodological strength of this work is the standardized probabilistic representation of established genotypic resistance criteria. Importantly, the present framework should not be viewed as a replacement for established rule-based resistance interpretation systems, which remain appropriate for well-characterized HBV resistance pathways [4,8,9]. Rather, its added value lies in providing a standardized probabilistic framework that can be externally validated and quantitatively evaluated using discrimination, calibration, and threshold-dependent performance metrics. Such an approach may be particularly relevant in settings involving heterogeneous sequence repositories, evolving resistance patterns, or future integration with phenotypic and longitudinal clinical data. Consistent with this interpretation, direct comparison with a deterministic rule-based classifier based on the same predefined mutation criteria demonstrated near-equivalent classification performance, as expected from the construction of the genotypic endpoint. Although the present model was not designed for direct therapeutic decision-making, the proposed approach may still have practical utility in research and surveillance contexts. Standardized probabilistic interpretation of HBV sequence data could support large-scale screening of publicly available repositories and assist in identifying sequence patterns that warrant further expert evaluation. Similar frameworks could potentially be incorporated into sequencing-analysis pipelines or sequence-analysis workflows to support standardized resistance interpretation alongside established expert-guideline approaches. In addition, quantitative modeling approaches may facilitate more consistent comparison of resistance-associated profiles across heterogeneous datasets and provide a structured basis for future integration with phenotypic resistance data, treatment history, or longitudinal clinical observations [11,12,13,14]. Although current HBV resistance interpretation appropriately relies on established rule-based systems, such approaches do not inherently provide calibrated probabilities or threshold-dependent performance estimates. In clinical and laboratory settings, probabilistic outputs primarily provide a quantitative representation of predefined genotypic criteria and allow formal evaluation of calibration and threshold-dependent behavior across datasets. Importantly, the model is not intended to guide treatment decisions in isolation. Accordingly, the reported discrimination, calibration, and threshold-based metrics should be interpreted as measures of reproducibility and probabilistic consistency within a predefined genotypic framework rather than direct evidence of independent clinical predictive utility. Treatment selection for chronic HBV infection remains dependent on clinical history, prior nucleos(t)ide analogue exposure, viral load kinetics, adherence, liver disease stage, and guideline-based therapeutic recommendations [2,3,4]. The clinical utility of the present framework therefore lies in structured sequence interpretation and reproducible resistance pathway screening, rather than direct therapeutic decision-making.

The use of logistic regression was deliberate. Although more complex machine learning methods are increasingly applied in biomedical and viral sequence analysis, black-box models can be difficult to interpret and may perform poorly when transported across datasets [10,11,12,16,18]. In contrast, logistic regression provides interpretable coefficients, calibrated probabilities, and straightforward evaluation of discrimination, calibration, and decision-analytic performance. Furthermore, this analytical approach incorporates established genotypic interpretation frameworks [22,23,24], robust statistical metrics for risk model calibration [25] and net benefit [26], standardized reporting and risk-of-bias evaluation tools [27,28,29,30]. This is aligned with recent recommendations emphasizing transparency, external validation, calibration, and full reporting of prediction models using regression or machine learning methods [18,31,32,33,34,35]. The present study therefore illustrates that methodological rigor may be more important than algorithmic complexity when the biological target is well defined.

Decision curve analysis further supported the potential utility of model-based classification across a range of threshold probabilities. This is relevant because discrimination metrics alone do not indicate whether a model is useful under plausible decision thresholds [13,14,18]. In the present context, decision curve analysis should be interpreted cautiously, because the outcome is a genotypic pathway proxy rather than a clinical endpoint. Nevertheless, it provides a useful framework for evaluating whether probabilistic classification could offer benefit compared with default strategies such as classifying all or no sequences as pathway-positive.

This study has limitations. First, the outcome was a genotypic proxy rather than phenotypic resistance, virological breakthrough, or clinical treatment failure. Consequently, external validation in HBVdb should be interpreted primarily as assessment of methodological reproducibility and transportability across independently processed sequence repositories rather than validation against clinical treatment outcomes. Second, public sequence repositories are vulnerable to incomplete metadata, uneven geographic representation, heterogeneous sequencing practices, and selective deposition. Publicly available HBV sequence repositories contain limited standardized clinical metadata, restricting robust subgroup analyses according to disease phase, viral load, cirrhosis status, treatment history, or other clinically relevant host-level characteristics. Third, although external validation was performed using an independent repository, further validation in prospectively collected clinical cohorts would be required before clinical implementation. Additional validation across geographically diverse populations, alternative sequencing platforms, and clinically annotated cohorts would further strengthen assessment of model transportability and robustness. Fourth, the feature panel was intentionally limited to known resistance-associated and contextual RT positions; therefore, the model was not designed to discover novel or emerging resistance patterns. Because feature selection was limited to predefined RT positions, the model may not capture previously unrecognized sequence patterns associated with antiviral resistance. Lastly, because the outcome definition was partly derived from established resistance-associated substitutions included within the predefined feature panel, the framework should be interpreted primarily as a probabilistic formalization of a known biological resistance pathway rather than de novo discovery of novel resistance mechanisms.

In conclusion, this study provides a transparent and externally validated framework for probabilistic identification of a biologically established entecavir resistance pathway in HBV polymerase sequences. By combining motif-anchored RT numbering, prespecified feature extraction, probability calibration, threshold evaluation, and independent external validation, the approach connects established HBV resistance biology with contemporary standards for trustworthy prediction modeling. Future work should evaluate whether similar frameworks can be extended to other HBV resistance pathways, integrated with phenotypic or longitudinal treatment-response data, and validated in clinically annotated cohorts.

## 5. Conclusions

In summary, this study presents a transparent and externally validated probabilistic framework for reconstructing a predefined entecavir resistance pathway from HBV polymerase sequences derived from publicly available databases. The observed performance primarily reflects the biologically structured nature of the predefined genotypic resistance definition and the consistency of the analytical pipeline across independent repositories. Rather than serving as an independent predictor of clinical resistance outcomes, the framework provides a reproducible probabilistic formalization of established resistance-associated sequence patterns. These findings support the feasibility of applying transparent and externally validated analytical approaches to standardized sequence-based resistance interpretation.

## Data Availability

The datasets analyzed during the current study are publicly available from the NCBI Nucleotide/GenBank database and HBVdb. The scripts used for data acquisition, preprocessing, feature extraction, model development, and evaluation are provided in the Supplementary Materials. All data generated from the analyses are included in this published article and its Supplementary Information files. Additional workflow details are available from the corresponding author on reasonable request.

## Supplementary Materials

The following supporting information can be downloaded at: https://www.mdpi.com/article/10.3390/v18060610/s1, Figure S1: Flow diagram of development dataset assembly; Figure S2: Reverse transcriptase numbering and feature extraction; Figure S3: Internal threshold-based performance evaluation; Table S1: Reverse transcriptase feature panel and amino-acid distributions; Table S2: Prevalence of resistance pathway components; Table S3: Comparative classification and probabilistic performance metrics across internal and external validation datasets; Supplementary Sections S1–S7: Detailed description of data acquisition, feature engineering, model development, calibration, internal validation, external validation, and generalization assessment; Supplementary Code: Computational scripts used for data acquisition, feature engineering, model development, evaluation, calibration, and external validation.

## Abbreviations

The following abbreviations are used in this manuscript:

AA	Amino acid
AI	Artificial intelligence
APC	Article processing charge
AUC	Area under the curve
EASL	European Association for the Study of the Liver
ETV	Entecavir
HBV	Hepatitis B virus
HBVdb	Hepatitis B Virus Database
NCBI	National Center for Biotechnology Information
PR	Precision–recall
ROC	Receiver operating characteristic
RT	Reverse transcriptase
TRIPOD-AI	Transparent Reporting of a multivariable prediction model for Individual Prognosis Or Diagnosis-Artificial Intelligence
WHO	World Health Organization
YMDD	Tyrosine-Methionine-Aspartate-Aspartate motif
