# Supplementary material for: Externally Validated Probabilistic Modeling of a Predefined Entecavir Resistance Pathway in HBV Using Independent Public Repositories
Source: Viruses. 2026 May 27;18(6):610. doi: 10.3390/v18060610 (PMC13308312; doi:10.3390/v18060610)
Supplement: Supplementary file 1 [file viruses-18-00610-s001.zip › viruses-4310476-supplementary.pdf]

# S1. Introduction

This supplementary section provides a detailed description of the computational steps used for model development, internal validation, and external validation. All analyses were conducted using custom Python scripts executed sequentially. The workflow was designed to ensure transparency, reproducibility, and strict separation between model development and validation phases, in line with TRIPOD-AI recommendations.

# S2. Data acquisition and cohort assembly for model development

## S2.1. Rationale

The reliability of sequence-based prediction models depends critically on transparent data acquisition and explicit documentation of inclusion and exclusion steps. Public viral sequence repositories contain heterogeneous submissions that differ in annotation quality, completeness, and redundancy. To minimize bias and to ensure reproducibility, we implemented a prespecified, script-based data acquisition and curation strategy that systematically filtered, quality-controlled, and deduplicated HBV polymerase sequences prior to model development.

## S2.2. NCBI sequence retriva

HBV polymerase coding sequences were retrieved from the NCBI Nucleotide database using Script 1 - Download NCBI HBV polymerase CDS (GenBank format), located in Code Section. This script executed a programmatic query designed to capture all records annotated as hepatitis B virus polymerase or reverse transcriptase coding sequences. Records were downloaded in GenBank format to preserve structured feature annotations, including coding sequence boundaries and descriptive metadata. Automated retrieval ensured that the dataset could be regenerated without manual intervention.

## S2.3. Translation and quality control

Translation of coding sequences into amino-acid sequences and initial quality control were performed using Script 2 - Extract polymerase AA sequences, QC, deduplicate (reported in Code Section). For each GenBank record, the longest plausible polymerase translation was retained. Quality control filters were applied to exclude sequences outside the expected polymerase length range or containing an excessive proportion of ambiguous amino-acid characters. These criteria were selected to reduce the inclusion of truncated fragments and low-confidence translations that could compromise downstream feature extraction.

## S2.3. Deduplication and independence safeguards

Exact duplicate amino-acid sequences were identified using a cryptographic hash (SHA1) of the translated sequence and removed prior to model development. This step reduced overrepresentation of frequently submitted isolates and ensured that each unique polymerase sequence contributed equally to model training and evaluation. Sequence hashes were retained to enable subsequent exclusion of overlapping sequences during external validation.

## S2.4. Motif anchoring and sequence eligibility

Sequence eligibility for feature extraction was assessed using Script 3 - Build feature matrix + labels (ETV pathway proxy), freeze encoder (reported in Code Section). Sequences were required to contain a YMDD-family motif (Y[MVIL]DD), which anchors reverse transcriptase (RT) position 204 and enables biologically meaningful RT numbering across sequences of variable length. Sequences lacking this motif were excluded, as RT positions could not be reliably indexed.

## S2.5. Final development cohort

After translation, quality control, deduplication, and motif anchoring, the resulting curated dataset constituted the final development cohort used for feature engineering, model training, and internal validation. All filtering steps were prespecified and implemented prior to outcome labeling or model fitting.

To facilitate interpretation of the analytical pipeline and to provide a high-level overview of the study design, Figure S1 summarizes the complete computational workflow implemented in this study. The figure illustrates the sequential steps from public data acquisition to external validation, including sequence translation and quality control, motif-anchored reverse transcriptase numbering, feature extraction, model development and calibration, internal validation, and independent external validation using HBVdb. This overview highlights the prespecified separation between model development and validation phases and provides a visual guide to the scripts and analytical decisions described in detail in the Methods and Supplementary Material.

Figure S1. Flow diagram of development dataset assembly

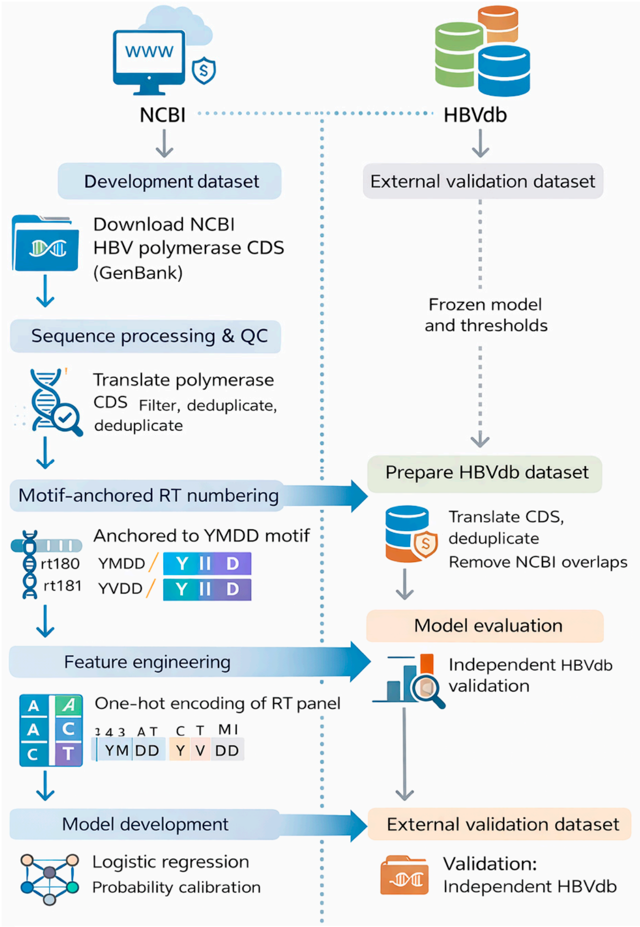


Flow diagram illustrating the assembly of the development dataset from the NCBI Nucleotide database. HBV polymerase coding sequences were retrieved in GenBank format, translated into amino-acid sequences, filtered by length and ambiguity, deduplicated using sequence hashes, and screened for the presence of a YMDD-family motif to enable reverse transcriptase numbering. Numbers indicate the count of sequences retained or excluded at each step.

To enhance transparency regarding the feature representation used for model development, the selected reverse transcriptase positions and the corresponding amino-acid distributions in the curated development and external validation cohorts are summarized in Supplementary Table S1. This table provides a descriptive overview of the residues most frequently observed at each position after quality control and deduplication and serves to document the biological plausibility and comparability of feature distributions across datasets.

Table S1. Reverse transcriptase feature panel and amino-acid distributions

| RT position | Functional relevance | Most frequent AA (NCBI) | Most frequent AA (HBVdb) |
|---|---|---|---|
| rt80 | Contextual position | L | L |
| rt84 | Contextual position | V | V |
| rt91 | Contextual position | I | I |
| rt169 | Contextual position | I | I |
| rt173 | Contextual position | V | V |
| rt180 | Lamivudine background | L | L |
| rt181 | Contextual position | A | A |
| rt184 | Entecavir-associated | T | T |
| rt191 | Contextual position | V | V |
| rt194 | Contextual position | A | A |
| rt200 | Contextual position | A | A |
| rt202 | Entecavir-associated | S | S |
| rt204 | YMDD motif anchor | M | M |
| rt215 | Contextual position | Q | Q |
| rt233 | Contextual position | I | I |
| rt236 | Contextual position | N | N |
| rt250 | Entecavir-associated | M | M |
| rt256 | Contextual position | C | C |
| rt269 | Contextual position | I | I |

Reverse transcriptase (RT) numbering is anchored to the conserved YMDD-family motif (Y[MVIL]DD), with the second residue corresponding to RT position 204. Amino acids are reported using standard one-letter codes. Frequencies reflect the most common residue observed at each RT position within the curated development cohort derived from NCBI GenBank and the independent external validation cohort derived from HBVdb, after quality control and deduplication. RT positions were selected a priori based on established associations with nucleos(t)ide analogue resistance or local sequence context.

Abbreviations: RT=reverse transcriptase; AA=amino acid; HBV=hepatitis B virus; NCBI=National Center for Biotechnology Information; HBVdb=Hepatitis B Virus Database; YMDD=tyrosine-methionine-aspartate-aspartate motif.

Amino-acid codes: A=Alanine; C=Cysteine; D=Aspartate; E=Glutamate; F=Phenylalanine; G=Glycine; I=Isoleucine; L=Leucine; M=Methionine; N=Asparagine; Q=Glutamine; S=Serine; T=Threonine; V=Valine; Y=Tyrosine.

To further characterize the construction of the outcome variable used for model development and validation, the prevalence of individual resistance pathway components is summarized in Supplementary Table S2. This table reports the frequency of lamivudine-associated background substitutions, entecavir-associated substitutions, and the complete entecavir resistance pathway in the development and external validation cohorts, thereby illustrating differences in pathway prevalence and compositional structure across datasets.

Table S2. Prevalence of resistance pathway components

| Criterion | Development cohort (%) | External cohort (%) |
|---|---|---|
| Lamivudine resistance background (rt180M + rt204V/I) | ~23% | ~2% |
| ≥1 entecavir-associated substitution (rt184 / rt202 / rt250) | ~23% | ~99% |
| Complete entecavir resistance pathway | 22.8% | 2.2% |

Resistance pathway components were defined based on established genotypic models of hepatitis B virus reverse transcriptase. A lamivudine resistance background was defined by the presence of substitutions at RT positions 180 and/or 204, while entecavir-associated substitutions were defined as the presence of at least one substitution at RT positions 184, 202, or 250. The complete entecavir resistance pathway required the coexistence of a lamivudine-associated background and at least one entecavir-associated substitution. Percentages reflect the proportion of

sequences meeting each criterion within the curated development cohort derived from NCBI GenBank and the independent external validation cohort derived from HBVdb.
Abbreviations: RT=reverse transcriptase; HBV=hepatitis B virus; ETV=entecavir; NCBI=National Center for Biotechnology Information; HBVdb=Hepatitis B Virus Database.

To further contextualize the relationship between the probabilistic modeling framework and the predefined genotypic endpoint definition, an additional comparator analysis was performed using a deterministic rule-based classifier directly implementing the same resistance-associated mutation criteria used for outcome construction. Comparative performance metrics for the rule-based and calibrated logistic regression approaches across the internal and external validation datasets are summarized in Supplementary Table S3. Threshold optimization procedures were predefined prior to external validation, and the selected operating threshold was subsequently frozen and applied unchanged to the HBVdb external cohort.

Table S3. Comparative classification and probabilistic performance metrics across internal and external validation datasets

**Classification performance comparison between the deterministic rule-based classifier and the calibrated logistic regression framework.**

| Dataset: | Method: | Threshold: | Accuracy: | Sensitivity: | Specificity: | PPV (Precision): | NPV: | F1: | MCC: | Concordance with rule: |
|---|---|---|---|---|---|---|---|---|---|---|
| **NCBI internal** | Rule-based classifier | N/A | 1.000 | 1.000 | 1.000 | 1.000 | 1.000 | 1.000 | 1.000 | N/A |
| **NCBI internal** | Logistic regression | 0.500 | 1.000 | 1.000 | 1.000 | 1.000 | 1.000 | 1.000 | 1.000 | 1.000 |
| **NCBI internal** | Logistic regression | 0.901 | 1.000 | 1.000 | 1.000 | 1.000 | 1.000 | 1.000 | 1.000 | 1.000 |
| **HBVdb external** | Rule-based classifier | N/A | 1.000 | 1.000 | 1.000 | 1.000 | 1.000 | 1.000 | 1.000 | N/A |
| **HBVdb external** | Logistic regression | 0.500 | 1.000 | 1.000 | 1.000 | 0.992 | 1.000 | 0.996 | 0.996 | 1.000 |
| **HBVdb external** | Logistic regression | 0.901 | 0.998 | 0.923 | 1.000 | 1.000 | 0.998 | 0.960 | 0.960 | 0.998 |

**Probabilistic performance metrics of the calibrated logistic regression framework across internal and external validation datasets.**

| Dataset: | Threshold: | AUC-ROC: | AUC-PR: | Brier Score: |
|---|---|---|---|---|
| NCBI internal | 0.500 | 1.0000 | 1.000 | 0.001 |
| NCBI internal | 0.901 | 1.0000 | 1.000 | 0.001 |
| HBVdb external | 0.500 | 1.0000 | 1.000 | 0.001 |
| HBVdb external | 0.901 | 1.0000 | 1.000 | 0.001 |

The deterministic rule-based classifier directly implemented the predefined resistance-associated mutation criteria used for endpoint construction. Logistic regression performance metrics are reported after application of the prespecified probability thresholds. The threshold of 0.901 corresponded to the optimal threshold identified during internal validation based on combined discrimination performance. Concordance with rule represents agreement between probabilistic model classifications and the deterministic rule-based classifier.
Abbreviations: AUC-PR=area under the precision–recall curve; AUC-ROC=area under the receiver operating characteristic curve; F1=harmonic mean of precision and sensitivity; HBVdb=Hepatitis B Virus Database; MCC=Matthews correlation coefficient; N/A=not applicable; RT=reverse transcriptase. TP=true positives; TN, true negatives; FP, false positives; FN, false negatives; NPV=negative predictive value; Accuracy=(TP + TN)/(TP + TN + FP + FN); Sensitivity=TP/(TP + FN); Specificity=TN/(TN + FP); Precision=TP/(TP + FP); F1=2 × [(Precision × Sensitivity)/(Precision + Sensitivity)]; MCC=[(TP × TN) − (FP × FN)] / √[(TP + FP)(TP + FN)(TN + FP)(TN + FN)]; NPV=TN / (TN + FN); Brier score=mean squared difference between predicted probabilities and observed outcomes.

## S3. Feature engineering and outcome definition

Following data acquisition and cohort assembly (described in section 1), the next step was to derive a feature representation of HBV polymerase sequences that preserves biological interpretability while enabling stable model training. This section focuses specifically on reverse transcriptase (RT) numbering,

feature construction, and outcome labeling, building directly on the curated sequence set generated in the preceding step.

## S3.1. Reverse transcriptase numbering

As introduced in Section 1, reverse transcriptase numbering was anchored using the conserved YMDD-family motif (Y[MVIL]DD), with RT position 204 defined by the second residue of the motif. Using Script 3, RT positions were indexed relative to this anchor, allowing consistent extraction of biologically meaningful residues across sequences of variable length. Sequences lacking a recognizable YMDD-family motif were excluded at this stage, as previously described.

## S3.2. RT feature panel construction

A predefined panel of RT positions was selected a priori based on established knowledge of HBV nucleos(t)ide analogue resistance. This panel included positions associated with lamivudine resistance background (e.g., rt180 and rt204), positions implicated in entecavir resistance (e.g., rt184, rt202, and rt250), and additional nearby positions included to provide local sequence context. The feature panel was fixed prior to model development to avoid data-driven selection and to preserve interpretability. For each sequence, the amino acid observed at each selected RT position was extracted and treated as a categorical feature. No substitution scores, evolutionary weights, or alignment-based similarity measures were applied.

## S3.3. Feature encoding

Categorical amino-acid features were transformed using one-hot encoding, implemented within Script 3. The encoder was fitted exclusively on the development dataset and subsequently frozen. This frozen encoder was reused unchanged for internal evaluation and external validation, ensuring consistent feature representation and preventing information leakage between datasets.

## S3.4. Final analytical dataset

The resulting analytical dataset consisted of one-hot encoded RT features, a binary outcome label, and associated metadata for each sequence. No additional features were introduced beyond this point. This dataset formed the sole input for model training and internal validation, as described in the subsequent supplementary section.

Figure S2. Reverse transcriptase numbering and feature extraction

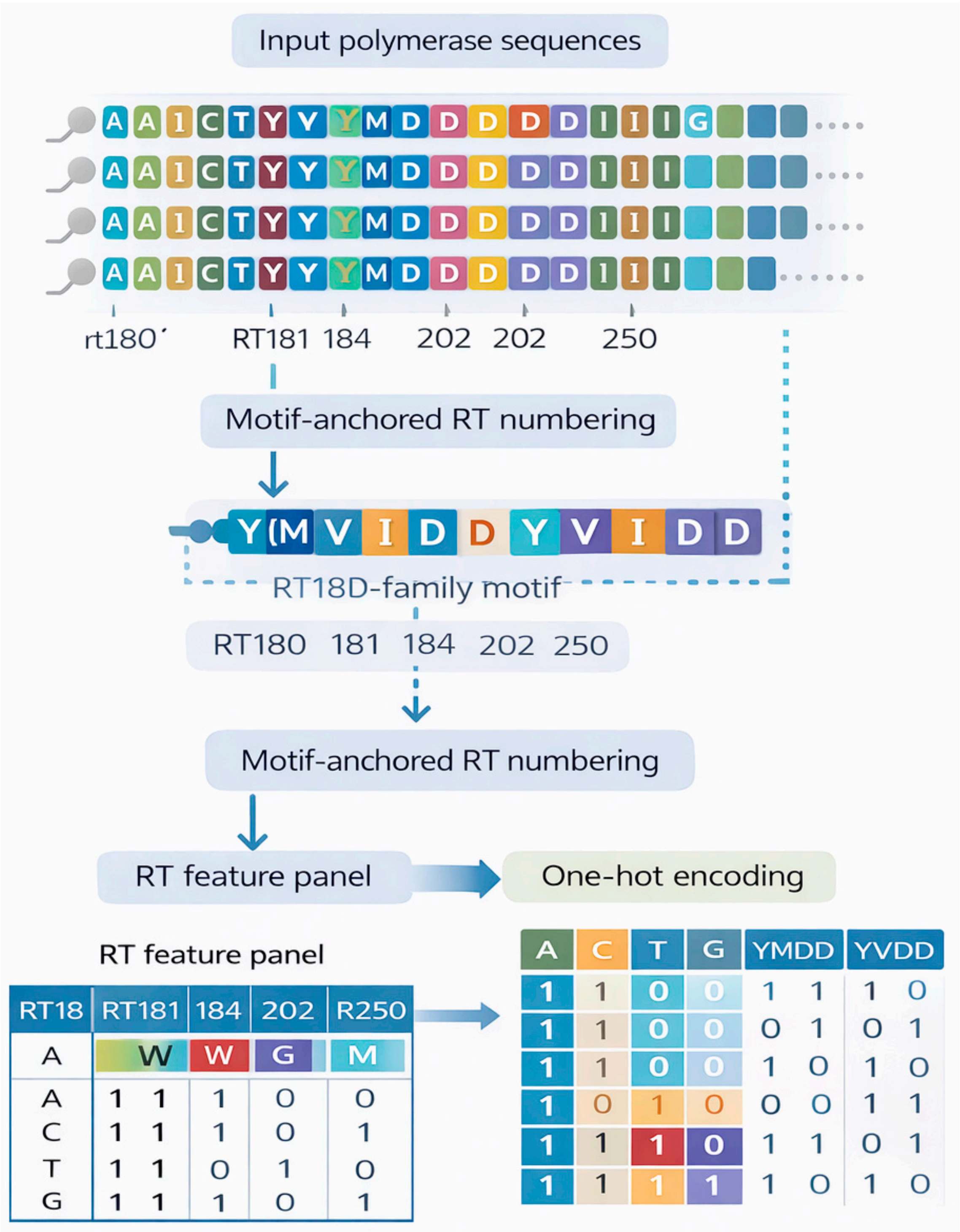


Illustration of motif-anchored reverse transcriptase numbering and feature extraction. RT position 204 was defined relative to the YMDD-family motif, enabling consistent indexing of resistance-associated positions across polymerase sequences of variable length. Amino acids at predefined RT positions were extracted and encoded as categorical features.

## S4. Model development, calibration, and internal validation

Following feature construction and outcome definition (described in section 2), model development and internal validation were performed to quantify the relationship between reverse transcriptase features and the predefined entecavir resistance pathway proxy. Given the mechanistic nature of the outcome and the categorical structure of the features, priority was given to model transparency, interpretability, and reproducibility over algorithmic complexity.

## S4.1. Model specification and training

Model training was implemented using Script 4 - Training and Internal validation of model (described in Code Section). A logistic regression classifier was selected as the primary modeling approach, as it provides direct probabilistic outputs and enables straightforward interpretation of feature contributions. This choice was made a priori and was not informed by comparative performance screening.

To address class imbalance in the development dataset, class weights were applied during model fitting. Model training was restricted to the development dataset, using a stratified train/test split to preserve outcome prevalence in the internal test subset. No sequences from the external validation cohort were used during this stage.

## S4.2. Probability calibration

Because predicted probabilities were intended to support threshold-based interpretation and decision-analytic evaluation, probability calibration was performed after model training using sigmoid calibration. Calibration was fitted exclusively on the training portion of the development dataset and applied unchanged to the internal test set. This step ensured that predicted probabilities more accurately reflected the observed frequency of the resistance pathway within the development cohort.

## S4.3. Internal validation strategy

Internal validation was conducted on the held-out test subset using Script 4, with performance assessed using both threshold-independent and threshold-dependent metrics. Discrimination was evaluated using the area under the receiver operating characteristic curve and the precision-recall curve, while calibration was assessed graphically and using the Brier score. Three decision thresholds were evaluated:

- A fixed threshold of 0.5
- A threshold maximizing the Youden index
- A threshold maximizing the F1 score.

The Youden-based threshold was prespecified as the primary operating point and selected using only internal validation data. This threshold was subsequently frozen and carried forward to external validation, which will be described in section 6.

## S4.4. Bootstrap uncertainty estimation

To quantify statistical uncertainty, internal validation metrics were supplemented with 95% confidence intervals estimated using nonparametric bootstrap resampling. Bootstrap sampling was performed at the sequence level, with metrics recalculated across repeated resamples. This approach provides an assessment of metric stability without relying on distributional assumptions.

## S4.5. Interpretation of internal performance

Internal validation yielded near-perfect discrimination and classification performance. This result was expected given that the outcome variable represents a deterministic genotypic pathway derived directly from a subset of the RT positions included in the feature set. Internal validation was therefore interpreted as a confirmation of correct feature extraction, encoding, and model implementation rather than as evidence of generalizable predictive performance. Accordingly, the primary objective of the framework was not discovery of novel resistance rules, but development of a calibrated and externally validated probabilistic implementation for standardized evaluation across independent sequence repositories. The primary assessment of generalization was reserved for external validation on an independent dataset, which will be described in sections 6 and 7.

## S4.6. Model freezing and artifact preservation

At the conclusion of internal validation, the trained logistic regression model, calibration parameters, feature encoder, and prespecified decision thresholds were serialized and frozen. These artifacts were

reused unchanged for all subsequent analyses, including external validation. No retraining, recalibration, or threshold optimization was performed beyond this point.

# S5. Model evaluation and threshold-based performance assessment

Following model training, calibration, and internal validation (described in previous sections), a dedicated evaluation step was performed to generate standardized performance summaries and diagnostic outputs for reporting. Separating model evaluation from model training ensures reproducibility of reported results and allows performance metrics and figures to be regenerated without retraining or modifying model parameters.

## S5.1. Evaluation framework

Model evaluation was conducted using Script 5 - Evaluation of model (described in Code Section), which reloads the frozen model, calibration parameters, feature encoder, and prespecified thresholds generated during internal validation. No refitting, recalibration, or threshold optimization was performed within this script. This evaluation step was applied exclusively to the internal test subset defined during model development and was used to generate final internal performance tables and figures for reporting and quality control.

## S5.2. Performance metrics

Model performance was summarized using a comprehensive set of complementary metrics. Threshold-independent discrimination was assessed using the area under the receiver operating characteristic curve and the precision-recall curve. Threshold-dependent classification performance was evaluated using sensitivity (recall), specificity, positive predictive value (PPV), negative predictive value (NPV), precision, accuracy, F1 score, Matthews correlation coefficient, and confusion matrices. Calibration was assessed using calibration curves and the Brier score, providing insight into the agreement between predicted probabilities and observed outcome frequencies. All metrics were computed consistently with definitions prespecified prior to analysis.

## S5.3. Decision threshold reporting

Three decision thresholds were evaluated to characterize model behavior across different operating points:

- A fixed threshold of 0.5
- The Youden index–optimized threshold selected during internal validation
- The threshold maximizing the F1 score.

Consistent with the analysis plan described in section 3, the Youden-based threshold was designated as the primary operating point. The remaining thresholds were included as sensitivity analyses to illustrate robustness of performance to threshold choice.

## S5.4. Bootstrap confidence intervals

To accompany point estimates, 95% confidence intervals for all reported metrics were computed using nonparametric bootstrap resampling. Bootstrap resampling was performed at the sequence level, and metrics were recalculated across repeated resamples. This approach provides an empirical assessment of variability without reliance on asymptotic assumptions.

## S5.5. Reporting outputs

The evaluation script generated tabular summaries of performance metrics and a set of diagnostic figures, including receiver operating characteristic curves, precision-recall curves, calibration plots, and confusion matrices. These outputs were used to populate the internal validation results reported in the manuscript (figure 2: Panels A-D) and supplementary tables. Importantly, all reported internal results reflect the frozen model and thresholds established prior to external validation.

Figure S3. Internal threshold-based performance evaluation

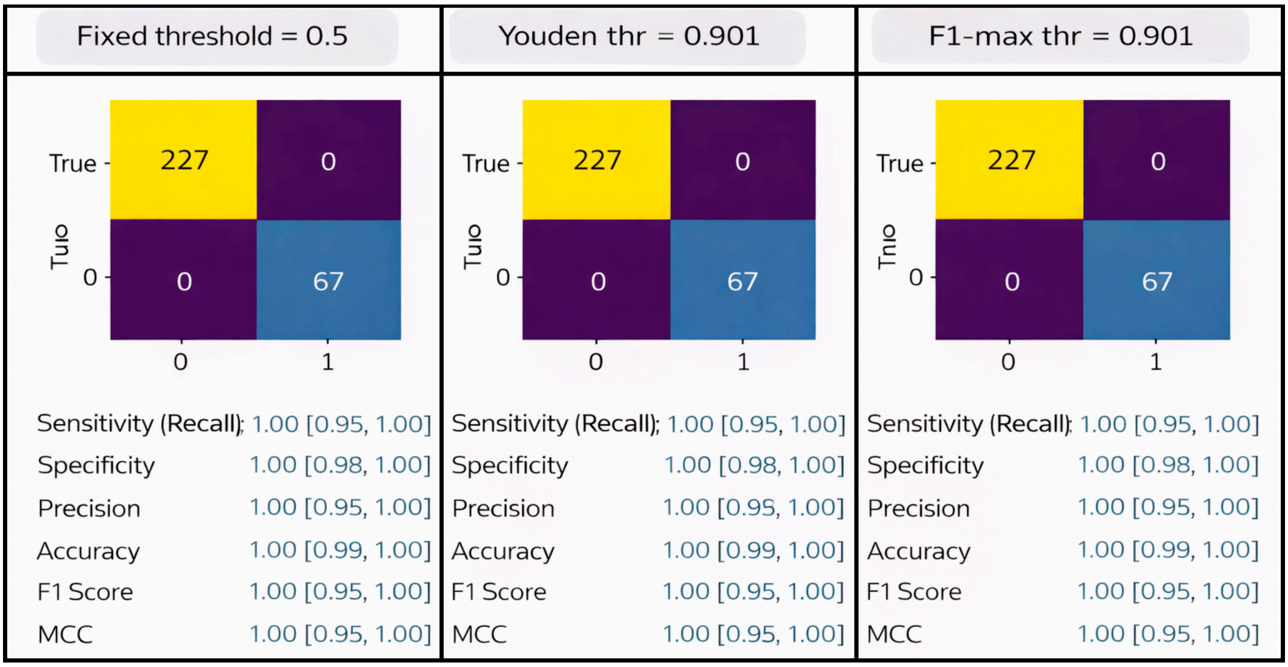

Threshold-based evaluation of the internally validated model. Confusion matrices and associated performance metrics are shown for a fixed probability threshold of 0.5, the internally optimized Youden threshold, and the F1-maximizing threshold. These analyses illustrate the effect of threshold selection on classification performance while preserving a prespecified primary operating point.

## S6. Preparation of the HBVdb cohort for external validation

External validation requires application of a trained model to a dataset that is independent of the development process and generated under different curation and submission workflows. To assess the generalizability of the proposed framework beyond the NCBI-derived development cohort, an external validation dataset was assembled from HBVdb, a curated hepatitis B virus sequence repository maintained independently of GenBank. All processing steps were designed to mirror the development pipeline where appropriate while preserving strict separation from model development.

### S6.1. Data source and retrieval

HBV polymerase nucleotide sequences were obtained from the HBVdb database and processed using Script 6 - Prepare HBVdb cohort for External validation (described in Code Section). Sequences corresponding to the polymerase (P) gene were downloaded in FASTA format. HBVdb was selected as an external source because it aggregates sequences from multiple studies, applies independent curation procedures, and differs from NCBI in submission pathways and annotation practices.

### S6.2. Translation and frame selection

Because HBVdb provides nucleotide-level sequences, polymerase amino-acid sequences were generated using six-frame translation. For each nucleotide sequence, candidate translations were evaluated based on sequence length and the presence of a YMDD-family motif (Y[MVIL]DD). The translated frame containing a valid motif and most consistent with the expected length of HBV polymerase was retained. Sequences for which no suitable translated frame could be identified were excluded from further analysis.

This procedure ensured that amino-acid sequences entering external validation were biologically compatible with the motif-anchored reverse transcriptase numbering framework used during model development.

### S6.3. Quality control

Quality control procedures applied to the HBVdb-derived amino-acid sequences mirrored those used for the development dataset (see section 1). Sequences outside the expected polymerase length range or

containing excessive ambiguity were excluded. Sequences lacking a recognizable YMDD-family motif were also excluded, as reverse transcriptase numbering could not be reliably assigned. This filtering step resulted in a high retention rate, with the vast majority of HBVdb sequences containing an identifiable RT204 anchor motif.

### S6.3. Deduplication against the development dataset

To ensure strict independence between development and external validation cohorts, translated HBVdb amino-acid sequences were deduplicated against the NCBI-derived development dataset using cryptographic sequence hashes generated during development cohort assembly (see section 1). Any HBVdb sequence with an identical amino-acid sequence to a development sequence was excluded from external validation. This procedure mitigates the risk of information leakage arising from cross-database redundancy.

### S6.4. Final external validation cohort

Following translation, quality control, motif screening, and deduplication, the final external validation cohort comprised 11.513 unique HBV polymerase amino-acid sequences, of which 248 (2.2%) met the predefined entecavir resistance pathway proxy. A small number of sequences were excluded due to absence of the anchor motif or quality control failure. No outcome labels, thresholds, model parameters, or calibration steps were applied during cohort preparation. The curated external cohort was exported as a polymerase amino-acid FASTA file and an accompanying metadata table and was used exclusively for external validation, as described in section 7.

## S7. External validation and generalization assessment

External validation represents the primary assessment of model generalizability and clinical relevance. Following completion of model development, calibration, internal validation, and independent cohort preparation (described in sections 3-5), the trained model was evaluated on the HBVdb-derived external cohort without retraining, recalibration, or modification of decision thresholds. This strategy was adopted to provide an unbiased estimate of performance under conditions that reflect real-world sequence heterogeneity and prevalence shifts.

### S7.1. External validation framework

External validation was performed using Script 7 - Model External validation using HBVdb database (described in Code Section). The script reloaded the frozen logistic regression model, probability calibration parameters, one-hot encoder, and prespecified decision thresholds established during internal validation. Feature extraction and encoding were applied identically to the external cohort, ensuring that the model was evaluated under the same representational assumptions used during development. No parameters were refitted, and no information from the external dataset was used to influence model structure, calibration, or threshold selection.

### S7.2. Performance assessment

Model performance on the external cohort was assessed using the same suite of metrics employed during internal evaluation to facilitate direct comparison. Discrimination was evaluated using the area under the receiver operating characteristic curve and the precision-recall curve. Threshold-dependent performance was summarized using sensitivity, specificity, precision, accuracy, F1 score, Matthews correlation coefficient, and confusion matrices at prespecified decision thresholds. Given the substantially lower prevalence of the entecavir resistance pathway in the external dataset, particular emphasis was placed on precision-recall analysis and threshold-based performance metrics.

## S7.3. Calibration assessment

Calibration of predicted probabilities was evaluated graphically using calibration curves and quantitatively using the Brier score. Calibration assessment was performed without recalibration, allowing direct evaluation of probability transportability from the development cohort to the external dataset. This approach assesses whether predicted probabilities retain their intended probabilistic interpretation under a different prevalence and data-generation context.

## S7.4. Decision curve analysis

To evaluate potential clinical utility, decision curve analysis was performed across a range of threshold probabilities. Net benefit was calculated for the model and compared with default "treat-all" and "treat-none" strategies. Decision curve analysis was included to complement traditional discrimination metrics and to assess whether the model provides meaningful benefit across clinically plausible operating thresholds.

## S7.5. Bootstrap confidence intervals

As in internal evaluation, external performance metrics were accompanied by 95% confidence intervals estimated using nonparametric bootstrap resampling at the sequence level. This approach provides an empirical assessment of metric stability in the external cohort without relying on asymptotic assumptions.

## S7.6. Interpretation of external validation results

External validation demonstrated preserved discrimination, calibration, and threshold-based performance despite a marked reduction in outcome prevalence relative to the development dataset. This finding supports the robustness of the feature representation, calibration strategy, and threshold selection. Importantly, external validation results were interpreted as evidence of generalization rather than optimization, as all analytical decisions were prespecified and frozen prior to evaluation.

---

# Code

### 1. Script 1 - Download NCBI HBV polymerase CDS (GenBank format)

**Description:** This script programmatically queries the NCBI Nucleotide database for hepatitis B virus records annotated with polymerase coding sequences and downloads the matching entries in GenBank format. Records are retrieved in batches to respect NCBI usage constraints and to reduce the likelihood of interrupted transfers. The output is a single GenBank file (ncbi/ncbi_pol.gb) that serves as the raw development corpus.
**Rationale:** Using a scripted NCBI query makes the data acquisition step transparent and reproducible: the search string, batch size, and retrieval method are explicitly documented and can be re-run by reviewers. Storing the results in GenBank format retains the feature annotations required to correctly identify coding sequences and translate them in downstream steps.
**Utility in the pipeline:** This is the entry point for the NCBI-based development dataset. All subsequent steps assume the presence of ncbi/ncbi_pol.gb.

**Script code:**

```
import os
import time
from Bio import Entrez

OUT_GB = "ncbi/ncbi_pol.gb"
QUERY = "Hepatitis B virus[Organism] AND polymerase[Title] AND cds[Title]"
BATCH = 200
SLEEP = 0.34

def chunks(lst, n):
    for i in range(0, len(lst), n):
        yield lst[i:i+n]
```

```
def main():
    os.makedirs("ncbi", exist_ok=True)

    Entrez.email = os.environ.get("NCBI_EMAIL", "christeloskapatais@hotmail.com")
    if not Entrez.email:
        raise SystemExit("Set NCBI_EMAIL environment variable")

    api_key = os.environ.get("NCBI_API_KEY")
    if api_key:
        Entrez.api_key = api_key

    h = Entrez.esearch(db="nucleotide", term=QUERY, retmax=0)
    r = Entrez.read(h)
    h.close()
    n = int(r["Count"])
    print(f"NCBI hits: {n}")

    h = Entrez.esearch(db="nucleotide", term=QUERY, retmax=n)
    r = Entrez.read(h)
    h.close()
    ids = r["IdList"]

    with open(OUT_GB, "w") as f:
        for batch in chunks(ids, BATCH):
            h = Entrez.efetch(db="nucleotide", id=",".join(batch), rettype="gb", retmode="text")
            f.write(h.read())
            f.write("\n")
            h.close()
            time.sleep(SLEEP)

    print(f"Wrote {OUT_GB}")

if __name__ == "__main__":
    main()
```

## 2. Script 2 - Extract polymerase AA sequences, QC, deduplicate

**Description:** This script parses the downloaded GenBank file and extracts polymerase coding sequences by scanning CDS features for polymerase-related keywords in the feature qualifiers (e.g., “polymerase”, “reverse transcriptase”, “pol”). For each record, it selects the longest plausible polymerase translation, applies quality control filters (protein length range and maximum fraction of ambiguous residues), and removes exact duplicates using a SHA1 hash of the amino-acid sequence. It exports:

1. An amino-acid FASTA file (ncbi/pol_aa.fasta)
2. A metadata table (ncbi/pol_meta.tsv) containing accession, length, hash, and description.

**Rationale:** Public sequence repositories contain fragments, truncated entries, and repeated submissions. Quality control and deduplication are therefore essential to prevent biased performance estimates and inadvertent information leakage across splits. The metadata table provides an auditable trail from the final analysis set back to the original accessions and facilitates later sensitivity analyses.

**Utility in pipeline:** This script converts raw GenBank entries into a curated amino-acid dataset suitable for motif-based indexing and feature engineering. It also produces the SHA1 hashes used later to remove overlaps between NCBI and HBVdb in the external validation stage.

**Script code:**

```
import os
import hashlib
import pandas as pd
from Bio import SeqIO

IN_GB = "ncbi/ncbi_pol.gb"
OUT_FASTA = "ncbi/pol_aa.fasta"
OUT_META = "ncbi/pol_meta.tsv"
```

```
MIN_AA = 500
MAX_AA = 1200
MAX_X_FRAC = 0.02
POL_KEYWORDS = ("polymerase", "reverse transcriptase", "pol")

def sha1(s: str) -> str:
    return hashlib.sha1(s.encode("utf-8")).hexdigest()

def is_pol_cds(feature) -> bool:
    if feature.type != "CDS":
        return False
    q = feature.qualifiers
    text = " ".join(q.get("product", []) + q.get("note", []) + q.get("gene", [])).lower()
    return any(k in text for k in POL_KEYWORDS)

def main():
    os.makedirs("ncbi", exist_ok=True)

    seen = set()
    rows = []
    kept = 0
    total = 0

    with open(OUT_FASTA, "w") as out_fa:
        for rec in SeqIO.parse(IN_GB, "genbank"):
            total += 1

            candidates = []
            for feat in rec.features:
                if not is_pol_cds(feat):
                    continue
                try:
                    nuc = feat.extract(rec.seq)
                    aa = str(nuc.translate(to_stop=False)).replace("*", "")
                    candidates.append((len(aa), aa))
                except Exception:
                    continue

            if not candidates:
                continue

            candidates.sort(reverse=True, key=lambda x: x[0])
            aa = candidates[0][1]

            if not (MIN_AA <= len(aa) <= MAX_AA):
                continue

            if aa.count("X") / len(aa) > MAX_X_FRAC:
                continue

            h = sha1(aa)
            if h in seen:
                continue

            seen.add(h)

            acc = rec.id
            out_fa.write(f">{acc}\n{aa}\n")

            rows.append(
                {
                    "accession": acc,
                    "aa_len": len(aa),
                    "sha1": h,
```

```
                "description": rec.description,
            }
        )

        kept += 1

    pd.DataFrame(rows).to_csv(OUT_META, sep="\t", index=False)

    print(f"GenBank records read: {total}")
    print(f"Polymerase AA kept: {kept}")
    print(OUT_FASTA)
    print(OUT_META)

if __name__ == "__main__":
    main()
```

## 3. Script 3 - Build feature matrix + labels (ETV pathway proxy), freeze encoder

**Description:** This script constructs the modeling dataset from the curated polymerase amino-acid FASTA. It first anchors reverse transcriptase (RT) numbering using the conserved YMDD-family motif, implemented as a regular expression Y[MVIL]DD, which accommodates wild-type (YMDD) and common resistance variants (e.g., YVDD, YIDD). After identifying the index corresponding to rt204 (the second residue in the motif), the script extracts residues at a predefined panel of RT positions. A binary label is then derived as a *genotypic proxy* for the entecavir-resistance pathway: lamivudine-associated background (rt180 = M and rt204 ∈ {V, I}) plus at least one additional substitution at rt184, rt202, or rt250 (as defined in the script). Feature values are one-hot encoded with an encoder saved to disk to ensure identical transformation during external validation. Outputs include a per-sequence table (results/dev_table.tsv), a compressed feature/label archive (results/dev_dataset.npz), and the frozen encoder (models/encoder.pkl).
**Rationale:** Two design choices are critical here. First, motif-anchored RT numbering is more robust than using absolute polymerase indices because HBV polymerase contains multiple domains and substantial variability in upstream sequence length. Second, freezing the one-hot encoder is necessary for external validation: it prevents any inadvertent re-learning of category levels from the external dataset and guarantees that the model receives a consistent feature representation across cohorts.
**Utility in pipeline:** This script bridges bioinformatics preprocessing and machine learning by producing the complete development dataset and the transformation objects required for reproducible inference on HBVdb.
**Script code:**

```
import os
import re
import numpy as np
import pandas as pd
import joblib
from Bio import SeqIO
from sklearn.preprocessing import OneHotEncoder

IN_FASTA = "ncbi/pol_aa.fasta"
OUT_NPZ = "results/dev_dataset.npz"
OUT_TABLE = "results/dev_table.tsv"
OUT_ENCODER = "models/encoder.pkl"

# RT positions (true rt-numbering) extracted using a motif anchor around rt204
RT_POS = [80, 84, 91, 169, 173, 180, 181, 184, 191, 194, 200, 202, 204, 215, 233, 236, 250, 256, 269]

# ETV pathway proxy definition (rt-numbering):
# LAM background: rt180=M AND rt204 in {V,I}
# AND ETV-associated: rt184 in set OR rt202 in set OR rt250=V
ETV_184_SET = {"A", "G", "I", "S", "L", "F", "M", "T"}
ETV_202_SET = {"G", "I"}

# Anchor motif family for rt204:
# Wild-type: YMDD
# LAM resistance: YVDD / YIDD
```

```
# Rare: YLDD
RT204_REGEX = re.compile(r"Y[MVIL]DD")

def find_rt204_index(seq: str) -> int | None:
    """
    Find index (0-based) of the rt204 residue within the YMDD-family motif.
    rt204 is the 2nd character in Y?DD.
    Example:
     YMDD -> rt204 is M
     YVDD -> rt204 is V
     YIDD -> rt204 is I
    """
    m = RT204_REGEX.search(seq)
    if m:
        return m.start() + 1
    return None

def residue_at_rt(seq: str, rt204_idx: int, rt_pos: int) -> str:
    """
    Return residue at given rt position using rt204 anchor.
    rt_pos 204 corresponds to seq[rt204_idx].
    """
    idx = rt204_idx + (rt_pos - 204)
    if idx < 0 or idx >= len(seq):
        return "-"
    return seq[idx]

def etv_pathway_label(calls: dict[int, str]) -> int:
    """
    Binary proxy label: Entecavir resistance pathway signature.
    Note: This is a genotypic proxy endpoint, not a clinical outcome label.
    """
    lam_bg = (calls.get(180, "-") == "M") and (calls.get(204, "-") in {"V", "I"})
    etv_spec = (
        (calls.get(184, "-") in ETV_184_SET) or
        (calls.get(202, "-") in ETV_202_SET) or
        (calls.get(250, "-") == "V")
    )
    return int(lam_bg and etv_spec)

def main() -> None:
    os.makedirs("results", exist_ok=True)
    os.makedirs("models", exist_ok=True)

    recs = list(SeqIO.parse(IN_FASTA, "fasta"))
    if not recs:
        raise SystemExit("No sequences found in ncbi/pol_aa.fasta. Run scripts 1–2 first.")

    ids: list[str] = []
    rows: list[list[str]] = []
    y: list[int] = []

    dropped_no_motif = 0

    for rec in recs:
        sid = rec.id.split()[0]
        seq = str(rec.seq)

        rt204_idx = find_rt204_index(seq)
        if rt204_idx is None:
            dropped_no_motif += 1
            continue
```

```
        calls = {p: residue_at_rt(seq, rt204_idx, p) for p in RT_POS}

        ids.append(sid)
        rows.append([calls[p] for p in RT_POS])
        y.append(etv_pathway_label(calls))

    df = pd.DataFrame(rows, columns=[f"rt{p}" for p in RT_POS])
    df.insert(0, "id", ids)
    df["y_etv_pathway"] = y
    df.to_csv(OUT_TABLE, sep="\t", index=False)

    enc = OneHotEncoder(handle_unknown="ignore", sparse_output=False)
    X = enc.fit_transform(df[[f"rt{p}" for p in RT_POS]].astype(str).values)

    np.savez_compressed(
        OUT_NPZ,
        X=X,
        y=np.array(y, dtype=int),
        ids=np.array(ids, dtype=object),
        rt_pos=np.array(RT_POS, dtype=int),
    )
    joblib.dump(enc, OUT_ENCODER)

    print(f"[✓] {OUT_TABLE}")
    print(f"[✓] {OUT_NPZ}")
    print(f"[✓] {OUT_ENCODER}")
    print(f"[i] Dropped (no Y[MVIL]DD motif found): {dropped_no_motif}")
    print(f"[i] Positives (ETV pathway): {int(np.sum(y))} / {len(y)}")

if __name__ == "__main__":
    main()
```

## 4. Script 4 – Training and Internal validation of model

**Description:** This script trains the primary predictive model and performs internal validation. The development dataset is split into training and test partitions using a stratified split. A logistic regression classifier is fitted with class weighting to address imbalance, and predicted probabilities are subsequently calibrated using sigmoid calibration (Platt scaling) via cross-validation. The script then evaluates model performance on the held-out internal test set using threshold-independent metrics (ROC AUC and PR AUC) as well as threshold-dependent metrics (accuracy, sensitivity/recall, specificity, precision, F1, MCC). It computes three decision thresholds: a fixed 0.5 threshold, a Youden's J (optimizing threshold), and an F1 (maximizing threshold). For each threshold, it estimates bootstrap 95% confidence intervals across metrics. The script saves the fitted models (models/etv_logreg.pkl, models/etv_logreg_calibrated.pkl), frozen thresholds (results/thresholds.json), a metrics table (results/internal_metrics_extended.tsv), and figures for ROC, PR, calibration, and decision curve analysis (DCA).

**Rationale:** Logistic regression provides a transparent baseline that is well-suited to sparse categorical features, while probability calibration supports clinically interpretable risk estimates. Freezing thresholds after internal optimization reduces optimism and prevents post hoc selection during external testing. Bootstrapped confidence intervals quantify uncertainty without relying on large-sample normal approximations, and the inclusion of calibration and DCA aligns the evaluation with contemporary reporting expectations for clinical prediction models.

**Utility in pipeline:** This script produces the final model artifact and the "internal validation package" (metrics, plots, thresholds) that define the prespecified operating points for external validation on HBVdb.

**Script code:**

```
import os, json
import numpy as np
import pandas as pd
import joblib
import matplotlib.pyplot as plt

from sklearn.model_selection import train_test_split
from sklearn.linear_model import LogisticRegression
from sklearn.calibration import CalibratedClassifierCV, calibration_curve
```

```
from sklearn.metrics import (
    roc_auc_score, average_precision_score,
    accuracy_score, f1_score, precision_score, recall_score,
    confusion_matrix, matthews_corrcoef, brier_score_loss
)

DATA = "results/dev_dataset.npz"

OUT_MODEL = "models/etv_logreg.pkl"
OUT_CAL_MODEL = "models/etv_logreg_calibrated.pkl"
OUT_THRESH = "results/thresholds.json"
OUT_METRICS = "results/internal_metrics_extended.tsv"

FIG_ROC = "figures/internal_roc.png"
FIG_PR = "figures/internal_pr.png"
FIG_CAL = "figures/internal_calibration.png"
FIG_DCA = "figures/internal_decision_curve.png"

BOOTSTRAPS = 2000
SEED = 42

def confusion_parts(y_true, y_pred):
    tn, fp, fn, tp = confusion_matrix(y_true, y_pred).ravel()
    return tn, fp, fn, tp

def specificity(y_true, y_pred) -> float:
    tn, fp, fn, tp = confusion_parts(y_true, y_pred)
    return tn / (tn + fp) if (tn + fp) else np.nan

def ece_score(y_true, y_prob, n_bins=10) -> float:
    bins = np.linspace(0, 1, n_bins + 1)
    ece = 0.0
    for i in range(n_bins):
        lo, hi = bins[i], bins[i+1]
        mask = (y_prob >= lo) & (y_prob < hi) if i < n_bins-1 else (y_prob >= lo) & (y_prob <= hi)
        if mask.sum() == 0:
            continue
        conf = float(y_prob[mask].mean())
        acc = float(y_true[mask].mean())
        ece += (mask.sum() / len(y_prob)) * abs(acc - conf)
    return float(ece)

def compute_metrics(y_true, y_prob, thr: float) -> dict:
    y_pred = (y_prob >= thr).astype(int)
    tn, fp, fn, tp = confusion_parts(y_true, y_pred)
    return {
        "threshold": float(thr),
        "AUC_ROC": roc_auc_score(y_true, y_prob) if len(np.unique(y_true)) == 2 else np.nan,
        "AUC_PR": average_precision_score(y_true, y_prob) if len(np.unique(y_true)) == 2 else np.nan,
        "accuracy": accuracy_score(y_true, y_pred),
        "sensitivity_recall": recall_score(y_true, y_pred, zero_division=0),
        "specificity": specificity(y_true, y_pred),
        "precision": precision_score(y_true, y_pred, zero_division=0),
        "F1": f1_score(y_true, y_pred, zero_division=0),
        "MCC": matthews_corrcoef(y_true, y_pred) if len(np.unique(y_pred)) > 1 else 0.0,
        "brier": brier_score_loss(y_true, y_prob),
        "ECE_10bin": ece_score(y_true, y_prob, 10),
        "n": int(len(y_true)),
        "pos": int(y_true.sum()),
        "tn": int(tn), "fp": int(fp), "fn": int(fn), "tp": int(tp),
    }
```

```
def bootstrap_ci(y_true, y_prob, thr: float, n_boot=2000, seed=42) -> dict:
    rng = np.random.default_rng(seed)
    n = len(y_true)
    keys =
["AUC_ROC","AUC_PR","accuracy","sensitivity_recall","specificity","precision","F1","MCC","brier","ECE_10bin"]
    samples = {k: [] for k in keys}

    for _ in range(n_boot):
        idx = rng.integers(0, n, size=n)
        yt = y_true[idx]
        yp = y_prob[idx]
        m = compute_metrics(yt, yp, thr)
        for k in keys:
            v = m[k]
            if np.isnan(v):
                continue
            samples[k].append(v)

    ci = {}
    for k in keys:
        arr = np.array(samples[k], dtype=float)
        if len(arr) < max(50, int(0.1*n_boot)):
            ci[k] = (np.nan, np.nan)
        else:
            ci[k] = (float(np.percentile(arr, 2.5)), float(np.percentile(arr, 97.5)))
    return ci

def find_thresholds(y_true, y_prob) -> dict:
    cand = np.unique(y_prob)
    if len(cand) > 2000:
        cand = np.quantile(y_prob, np.linspace(0, 1, 2001))


    best_youden_thr, best_youden = 0.5, -1e9
    best_f1_thr, best_f1 = 0.5, -1e9

    for thr in cand:
        y_pred = (y_prob >= thr).astype(int)
        sens = recall_score(y_true, y_pred, zero_division=0)
        spec = specificity(y_true, y_pred)
        youden = (sens + spec - 1.0) if not np.isnan(spec) else -1e9
        f1 = f1_score(y_true, y_pred, zero_division=0)
        if youden > best_youden:
            best_youden = youden
            best_youden_thr = float(thr)
        if f1 > best_f1:
            best_f1 = f1
            best_f1_thr = float(thr)

    return {
        "fixed_0.5": 0.5,
        "youdenJ": best_youden_thr,
        "f1_max": best_f1_thr,
        "youdenJ_value": float(best_youden),
        "f1_value": float(best_f1),
    }

def plot_roc(y_true, y_prob, path):
    from sklearn.metrics import roc_curve
    fpr, tpr, _ = roc_curve(y_true, y_prob)
    plt.figure(figsize=(6,5))
```

```python
    plt.plot(fpr, tpr)
    plt.plot([0,1],[0,1], linestyle="--")
    plt.xlabel("False Positive Rate"); plt.ylabel("True Positive Rate")
    plt.title("ROC (internal test split)")
    plt.tight_layout(); plt.savefig(path, dpi=200)

def plot_pr(y_true, y_prob, path):
    from sklearn.metrics import precision_recall_curve
    p, r, _ = precision_recall_curve(y_true, y_prob)
    plt.figure(figsize=(6,5))
    plt.plot(r, p)
    plt.xlabel("Recall"); plt.ylabel("Precision")
    plt.title("PR (internal test split)")
    plt.tight_layout(); plt.savefig(path, dpi=200)

def plot_cal(y_true, y_prob, path):
    frac_pos, mean_pred = calibration_curve(y_true, y_prob, n_bins=10, strategy="uniform")
    plt.figure(figsize=(6,5))
    plt.plot(mean_pred, frac_pos, marker="o")
    plt.plot([0,1],[0,1], linestyle="--")
    plt.xlabel("Mean predicted probability"); plt.ylabel("Fraction positive")
    plt.title("Calibration (internal test split)")
    plt.tight_layout(); plt.savefig(path, dpi=200)

def decision_curve(y_true, y_prob, pts):
    n = len(y_true)
    prev = y_true.mean()
    nb_model, nb_all = [], []
    nb_none = np.zeros_like(pts, dtype=float)

    for pt in pts:
        y_pred = (y_prob >= pt).astype(int)
        tn, fp, fn, tp = confusion_parts(y_true, y_pred)
        w = pt / (1-pt)
        nb_m = (tp/n) - (fp/n)*w
        nb_a = prev - (1-prev)*w
        nb_model.append(nb_m)
        nb_all.append(nb_a)

    return np.array(nb_model), np.array(nb_all), nb_none

def plot_dca(y_true, y_prob, path):
    pts = np.linspace(0.01, 0.99, 99)
    nb_m, nb_a, nb_n = decision_curve(y_true, y_prob, pts)
    plt.figure(figsize=(6.5,5))
    plt.plot(pts, nb_m, label="Model")
    plt.plot(pts, nb_a, linestyle="--", label="Treat-all")
    plt.plot(pts, nb_n, linestyle=":", label="Treat-none")
    plt.xlabel("Threshold probability"); plt.ylabel("Net benefit")
    plt.title("Decision Curve (internal test split)")
    plt.legend()
    plt.tight_layout(); plt.savefig(path, dpi=200)

def main():
    os.makedirs("models", exist_ok=True)
    os.makedirs("results", exist_ok=True)
    os.makedirs("figures", exist_ok=True)

    z = np.load(DATA, allow_pickle=True)
    X = z["X"]
    y = z["y"].astype(int)
```

```
    Xtr, Xte, ytr, yte = train_test_split(X, y, test_size=0.25, stratify=y, random_state=SEED)

    base = LogisticRegression(max_iter=2000, class_weight="balanced")
    base.fit(Xtr, ytr)
    joblib.dump(base, OUT_MODEL)

    cal = CalibratedClassifierCV(base, method="sigmoid", cv=5)
    cal.fit(Xtr, ytr)
    joblib.dump(cal, OUT_CAL_MODEL)

    prob = cal.predict_proba(Xte)[:, 1]

    thresholds = find_thresholds(yte, prob)
    with open(OUT_THRESH, "w") as f:
        json.dump(thresholds, f, indent=2)

    rows = []
    for tname in ["youdenJ", "fixed_0.5", "f1_max"]:
        thr = float(thresholds[tname])
        point = compute_metrics(yte, prob, thr)
        ci = bootstrap_ci(yte, prob, thr, n_boot=BOOTSTRAPS, seed=SEED)

        row = {"dataset": "NCBI_internal_test", "model": "calibrated_logreg", "threshold_name": tname}
        for k, v in point.items():
            if k in {"tn","fp","fn","tp"}:
                continue
            row[k] = v
        for k, (lo, hi) in ci.items():
            row[f"{k}_ci95_lo"] = lo
            row[f"{k}_ci95_hi"] = hi
        rows.append(row)

    pd.DataFrame(rows).to_csv(OUT_METRICS, sep="\t", index=False)

    if len(np.unique(yte)) == 2:
        plot_roc(yte, prob, FIG_ROC)
        plot_pr(yte, prob, FIG_PR)
    plot_cal(yte, prob, FIG_CAL)
    plot_dca(yte, prob, FIG_DCA)

    print(f"[✓] Model: {OUT_CAL_MODEL}")
    print(f"[✓] Thresholds: {OUT_THRESH}")
    print(f"[✓] Metrics: {OUT_METRICS}")
    print(f"[✓] Plots in figures/")

if __name__ == "__main__":
    main()
```

## 5. Script 5 – Evaluation of model

**Description:** This script provides a standalone, reproducible evaluation of the *saved* calibrated model on a fixed internal test split. It reloads the processed dataset and the trained model, computes predicted probabilities, and reports discrimination (ROC AUC, PR AUC), classification performance across the prespecified thresholds (Youden J, fixed 0.5, F1-max), and the confusion matrix components. It exports a CSV summary table (results/model_eval_internal.csv) and produces figures for ROC, precision/recall, calibration, and confusion matrices for each thresholding strategy.
**Rationale:** Separating evaluation from training makes the workflow easier to audit and simplifies manuscript preparation: figures and summary tables can be regenerated without refitting the model. The script is also useful as a "sanity-check" utility to confirm that model serialization, threshold files, and the deterministic split specification yield identical results across computing environments.
**Utility in pipeline:** This script is best viewed as the reporting layer for internal validation (particularly helpful when assembling supplementary material and ensuring reproducibility at the revision stage).
**Script code:**

```
import os
import json
import numpy as np
import pandas as pd
import joblib
import matplotlib.pyplot as plt

from sklearn.model_selection import train_test_split
from sklearn.metrics import (
    roc_auc_score, average_precision_score,
    accuracy_score, precision_score, recall_score, f1_score,
    confusion_matrix, matthews_corrcoef, brier_score_loss,
    roc_curve, precision_recall_curve
)
from sklearn.calibration import calibration_curve

DATA_NPZ = "results/dev_dataset.npz"
MODEL_PKL = "models/etv_logreg_calibrated.pkl"
THRESH_JSON = "results/thresholds.json"  # optional; if missing, uses 0.5 only

OUT_DIR_RESULTS = "results"
OUT_DIR_FIG = "figures"
OUT_CSV = "results/model_eval_internal.csv"

FIG_ROC = "figures/eval_internal_roc.png"
FIG_PR = "figures/eval_internal_pr.png"
FIG_CAL = "figures/eval_internal_calibration.png"
FIG_CM_05 = "figures/eval_internal_confusion_0.5.png"
FIG_CM_YOUDEN = "figures/eval_internal_confusion_youden.png"
FIG_CM_F1 = "figures/eval_internal_confusion_f1max.png"
# ------------------------------------------

SEED = 42
TEST_SIZE = 0.25
def specificity(y_true, y_pred) -> float:
    tn, fp, fn, tp = confusion_matrix(y_true, y_pred).ravel()
    return tn / (tn + fp) if (tn + fp) else np.nan

def metric_row(y_true: np.ndarray, y_prob: np.ndarray, thr: float, thr_name: str) -> dict:
    y_pred = (y_prob >= thr).astype(int)
    tn, fp, fn, tp = confusion_matrix(y_true, y_pred).ravel()

    row = {
        "threshold_name": thr_name,
        "threshold": float(thr),
        "n_test": int(len(y_true)),
        "pos_test": int(y_true.sum()),
        "tn": int(tn), "fp": int(fp), "fn": int(fn), "tp": int(tp),

        "AUC_ROC": roc_auc_score(y_true, y_prob) if len(np.unique(y_true)) == 2 else np.nan,
        "AUC_PR": average_precision_score(y_true, y_prob) if len(np.unique(y_true)) == 2 else np.nan,

        "accuracy": accuracy_score(y_true, y_pred),
        "sensitivity_recall": recall_score(y_true, y_pred, zero_division=0),
        "specificity": specificity(y_true, y_pred),
        "precision": precision_score(y_true, y_pred, zero_division=0),
        "F1": f1_score(y_true, y_pred, zero_division=0),
        "MCC": matthews_corrcoef(y_true, y_pred) if len(np.unique(y_pred)) > 1 else 0.0,

        "brier": brier_score_loss(y_true, y_prob),
    }
    return row
```

```python
def plot_confusion(y_true, y_pred, path, title):
    cm = confusion_matrix(y_true, y_pred)
    plt.figure(figsize=(5.2, 4.5))
    plt.imshow(cm)
    plt.title(title)
    plt.xlabel("Predicted")
    plt.ylabel("True")
    plt.xticks([0, 1], ["0", "1"])
    plt.yticks([0, 1], ["0", "1"])

    # annotate
    for (i, j), val in np.ndenumerate(cm):
        plt.text(j, i, str(val), ha="center", va="center")

    plt.tight_layout()
    plt.savefig(path, dpi=200)

def plot_roc_curve(y_true, y_prob, path):
    fpr, tpr, _ = roc_curve(y_true, y_prob)
    plt.figure(figsize=(6, 5))
    plt.plot(fpr, tpr)
    plt.plot([0, 1], [0, 1], linestyle="--")
    plt.xlabel("False Positive Rate")
    plt.ylabel("True Positive Rate")
    plt.title("ROC curve (internal test split)")
    plt.tight_layout()
    plt.savefig(path, dpi=200)

def plot_pr_curve(y_true, y_prob, path):
    p, r, _ = precision_recall_curve(y_true, y_prob)
    plt.figure(figsize=(6, 5))
    plt.plot(r, p)
    plt.xlabel("Recall")
    plt.ylabel("Precision")
    plt.title("Precision–Recall curve (internal test split)")
    plt.tight_layout()
    plt.savefig(path, dpi=200)

def plot_calibration(y_true, y_prob, path):
    frac_pos, mean_pred = calibration_curve(y_true, y_prob, n_bins=10, strategy="uniform")
    plt.figure(figsize=(6, 5))
    plt.plot(mean_pred, frac_pos, marker="o")
    plt.plot([0, 1], [0, 1], linestyle="--")
    plt.xlabel("Mean predicted probability")
    plt.ylabel("Fraction of positives")
    plt.title("Calibration curve (internal test split)")
    plt.tight_layout()
    plt.savefig(path, dpi=200)

def main():
    os.makedirs(OUT_DIR_RESULTS, exist_ok=True)
    os.makedirs(OUT_DIR_FIG, exist_ok=True)

    # Load data
    z = np.load(DATA_NPZ, allow_pickle=True)
    X = z["X"]
    y = z["y"].astype(int)

    # Reproducible internal split (same SEED each time)
    Xtr, Xte, ytr, yte = train_test_split(
        X, y, test_size=TEST_SIZE, random_state=SEED, stratify=y
    )
```

```
    # Load model
    clf = joblib.load(MODEL_PKL)
    y_prob = clf.predict_proba(Xte)[:, 1]

    # Load thresholds (optional)
    thresholds = {"fixed_0.5": 0.5}
    if os.path.exists(THRESH_JSON):
        with open(THRESH_JSON, "r") as f:
            thresholds.update(json.load(f))

    # Compute metrics table
    rows = []
    for name in ["youdenJ", "fixed_0.5", "f1_max"]:
        if name not in thresholds:
            continue
        thr = float(thresholds[name])
        rows.append(metric_row(yte, y_prob, thr, name))

    df = pd.DataFrame(rows)
    df.to_csv(OUT_CSV, index=False)

    print(f"[✓] Saved metrics CSV: {OUT_CSV}")
    print(df.to_string(index=False))

    # Plots (threshold-independent)
    if len(np.unique(yte)) == 2:
        plot_roc_curve(yte, y_prob, FIG_ROC)
        plot_pr_curve(yte, y_prob, FIG_PR)
        print(f"[✓] ROC: {FIG_ROC}")
        print(f"[✓] PR:  {FIG_PR}")

    plot_calibration(yte, y_prob, FIG_CAL)
    print(f"[✓] Calibration: {FIG_CAL}")

    y_pred_05 = (y_prob >= 0.5).astype(int)
    plot_confusion(yte, y_pred_05, FIG_CM_05, "Confusion matrix (threshold = 0.5)")
    print(f"[✓] Confusion 0.5: {FIG_CM_05}")

    # Youden
    if "youdenJ" in thresholds:
        thr = float(thresholds["youdenJ"])
        y_pred = (y_prob >= thr).astype(int)
        plot_confusion(yte, y_pred, FIG_CM_YOUDEN, f"Confusion matrix (Youden thr = {thr:.3f})")
        print(f"[✓] Confusion Youden: {FIG_CM_YOUDEN}")

    # F1 max
    if "f1_max" in thresholds:
        thr = float(thresholds["f1_max"])
        y_pred = (y_prob >= thr).astype(int)
        plot_confusion(yte, y_pred, FIG_CM_F1, f"Confusion matrix (F1-max thr = {thr:.3f})")
        print(f"[✓] Confusion F1-max: {FIG_CM_F1}")

    # Small extra: print confusion matrix values for quick inspection
    print("\n[i] Confusion matrix at 0.5:")
    print(confusion_matrix(yte, y_pred_05))

if __name__ == "__main__":
    main()
```

## 6. Script 6 – Prepare HBVdb cohort for External validation

**Description:** This script prepares the external validation cohort from HBVdb. It optionally downloads the HBVdb P-gene nucleotide FASTA dataset and then translates each nucleotide sequence into amino acids using a six-frame strategy (three forward frames plus three reverse-complement frames). The “best” translation is selected by prioritizing sequences containing the YMDD-family motif (Y[MVIL]DD) and, secondarily, longer translations. Quality control filters remove atypical lengths and sequences with excessive ambiguous residues. To ensure independence between development and external validation cohorts, the script removes sequences that match any NCBI development sequence by SHA1 hash of the translated amino-acid sequence. Outputs include the cleaned external amino-acid FASTA (hbvdb/pol_aa_external.fasta) and a metadata table (hbvdb/pol_external_meta.tsv) documenting translation strand/frame and motif presence.
**Rationale:** HBVdb entries may include sequences in different orientations or frames relative to the reference polymerase protein, and a six-frame approach improves robustness without requiring complex annotation parsing. Motif-informed selection increases the yield of RT-interpretable translations, which is essential for downstream RT-position extraction. Deduplication against NCBI is a key methodological safeguard: without it, “external validation” can be undermined by shared accessions between repositories.
**Utility in pipeline:** This script produces an external cohort that is both technically compatible with the feature engineering strategy and methodologically defensible as an independent validation set.
**Script code:**

```
import os
import re
import time
import hashlib
import argparse
import pandas as pd
from Bio import SeqIO
from Bio.Seq import Seq
from urllib.request import urlopen, Request
from urllib.error import URLError, HTTPError

# -------------------------
# INPUTS / OUTPUTS
# -------------------------
HBVDB_URL_ALL_P = "https://hbvdb.lyon.inserm.fr/data/nucleic/fasta/all_P.fas"
HBVDB_NUC = "hbvdb/all_P.fas"        # downloaded (or manually placed) HBVdb P gene nucleotide FASTA
NCBI_AA = "ncbi/pol_aa.fasta"        # development AA FASTA

OUT_AA = "hbvdb/pol_aa_external.fasta"
OUT_META = "hbvdb/pol_external_meta.tsv"

# -------------------------
# QC / MOTIF
# -------------------------
MIN_AA = 500
MAX_AA = 1200
MAX_X_FRAC = 0.02

# Anchor motif family for rt204 (covers WT + common LAM-resistant variants)
RT204_REGEX = re.compile(r"Y[MVIL]DD")

def sha1(s: str) -> str:
    return hashlib.sha1(s.encode("utf-8")).hexdigest()

def download_with_retries(url: str, out_path: str, retries: int = 8, backoff_sec: float = 2.0) -> None:
    """
    Download a file with retry/backoff to handle intermittent HBVdb 502 errors.
    Uses only Python stdlib (urllib) for portability.
    """
    os.makedirs(os.path.dirname(out_path), exist_ok=True)

    headers = {"User-Agent": "Mozilla/5.0 (HBVdb downloader for academic use)"}
    last_err = None
```

```
    for attempt in range(1, retries + 1):
        try:
            req = Request(url, headers=headers)
            with urlopen(req, timeout=60) as r, open(out_path, "wb") as f:
                # stream in chunks
                while True:
                    chunk = r.read(1024 * 1024)
                    if not chunk:
                        break
                    f.write(chunk)
            return
        except (HTTPError, URLError, TimeoutError) as e:
            last_err = e
            wait = backoff_sec * attempt
            print(f"[!] Download attempt {attempt}/{retries} failed: {e}. Retrying in {wait:.1f}s ...")
            time.sleep(wait)

    raise SystemExit(f"Failed to download after {retries} attempts. Last error: {last_err}\n"
                     f"Please download manually from HBVdb and save to: {out_path}")

def load_ncbi_hashes() -> set[str]:
    hashes = set()
    for rec in SeqIO.parse(NCBI_AA, "fasta"):
        hashes.add(sha1(str(rec.seq)))
    return hashes

def translate_seq(nuc: str, frame: int) -> str:
    aa = str(Seq(nuc[frame:]).translate(to_stop=False))
    return aa.replace("*", "")

def best_translation_with_motif(nuc: str) -> tuple[str, str, int, bool]:
    """
    6-frame translation (forward + reverse-complement, 3 frames each).
    Prefer candidates containing Y[MVIL]DD motif; then prefer longer length.
    Returns (aa, strand, frame, has_motif).
    """
    nuc = nuc.upper().replace(" ", "").replace("\n", "")
    candidates = []

    for frame in (0, 1, 2):
        aa = translate_seq(nuc, frame)
        candidates.append((aa, "+", frame, bool(RT204_REGEX.search(aa))))

    rc = str(Seq(nuc).reverse_complement())
    for frame in (0, 1, 2):
        aa = translate_seq(rc, frame)
        candidates.append((aa, "-", frame, bool(RT204_REGEX.search(aa))))

    candidates.sort(key=lambda x: (x[3], len(x[0])), reverse=True)
    return candidates[0]

def passes_qc(aa: str) -> bool:
    if not (MIN_AA <= len(aa) <= MAX_AA):
        return False
    if aa.count("X") / len(aa) > MAX_X_FRAC:
        return False
    return True

def main():
    parser = argparse.ArgumentParser(description="Prepare HBVdb external dataset (download optional), translate, QC,
dedup.")
    parser.add_argument("--download", action="store_true",
```

```
            help="Download HBVdb all_P.fas automatically into hbvdb/all_P.fas")
parser.add_argument("--force-download", action="store_true",
            help="Force re-download even if hbvdb/all_P.fas exists")
args = parser.parse_args()

os.makedirs("hbvdb", exist_ok=True)

# Optional download step
if args.download:
    if os.path.exists(HBVDB_NUC) and not args.force_download:
        print(f"[i] HBVdb file already exists: {HBVDB_NUC} (use --force-download to overwrite)")
    else:
        print(f"[i] Downloading HBVdb P-gene dataset from: {HBVDB_URL_ALL_P}")
        download_with_retries(HBVDB_URL_ALL_P, HBVDB_NUC)
        print(f"[✓] Downloaded to: {HBVDB_NUC}")

if not os.path.exists(HBVDB_NUC):
    raise SystemExit(
        f"Missing HBVdb input file: {HBVDB_NUC}\n"
        f"Either run with --download, or download manually and save it there."
    )

# Load development hashes for overlap removal
if not os.path.exists(NCBI_AA):
    raise SystemExit(f"Missing development AA file: {NCBI_AA} (run scripts 1–2 first).")

ncbi_hashes = load_ncbi_hashes()

kept = 0
dropped_overlap = 0
dropped_qc = 0

rows = []

with open(OUT_AA, "w") as out_fa:
    for rec in SeqIO.parse(HBVDB_NUC, "fasta"):
        sid = rec.id
        nuc = str(rec.seq)

        aa, strand, frame, has_motif = best_translation_with_motif(nuc)

        if not passes_qc(aa):
            dropped_qc += 1
            continue

        h = sha1(aa)
        if h in ncbi_hashes:
            dropped_overlap += 1
            continue

        out_fa.write(f">{sid}\n{aa}\n")
        rows.append({
            "id": sid,
            "aa_len": len(aa),
            "sha1": h,
            "strand": strand,
            "frame": frame,
            "has_rt204_motif": int(has_motif),
        })
        kept += 1

pd.DataFrame(rows).to_csv(OUT_META, sep="\t", index=False)

print(f"[✓] External AA kept: {kept}")
```

```
    print(f"[i] Dropped overlap vs NCBI (hash match): {dropped_overlap}")
    print(f"[i] Dropped QC: {dropped_qc}")
    print(f"[✓] Wrote {OUT_AA}")
    print(f"[✓] Wrote {OUT_META}")

    if rows:
        motif_rate = sum(r["has_rt204_motif"] for r in rows) / len(rows)
        print(f"[i] External sequences with Y[MVIL]DD motif: {motif_rate:.2%}")

if __name__ == "__main__":
    main()
```

## 7. Script 7 – Model External validation using HBVdb database

**Description:** This script executes external validation on HBVdb using the frozen preprocessing and model artifacts from the development stage. It reads the HBVdb amino-acid FASTA, anchors RT numbering via the same YMDD-family motif used in development, and extracts residues at the predefined RT position panel. Features are transformed using the previously saved one-hot encoder, and probabilities are generated using the calibrated logistic regression model. The script applies the prespecified thresholds (Youden J as primary; fixed 0.5 and F1-max as sensitivity analyses) and reports discrimination, threshold-dependent metrics, and calibration diagnostics (Brier score and an expected calibration error estimate). It exports an external metrics table (results/external_metrics_extended.tsv) and generates ROC, PR, calibration, and DCA figures.
**Rationale:** A valid external validation requires that no aspect of the preprocessing, model fitting, or threshold selection is re-optimized on the external dataset. By reusing the frozen encoder, calibrated model, and thresholds, this script enforces that separation. Reporting both discrimination and calibration (along with DCA) supports a more complete assessment of model transportability than AUC alone, particularly under the lower prevalence conditions typical of external repositories.
**Utility in pipeline:** This script produces the primary external validation results and figures that support claims of generalizability across repositories. It is also the key artifact reviewers can run to reproduce the external validation end-to-end.
**Script code:**

```
import os, json, re
import numpy as np
import pandas as pd
import joblib
import matplotlib.pyplot as plt

from Bio import SeqIO
from sklearn.metrics import (
    roc_auc_score, average_precision_score,
    accuracy_score, f1_score, precision_score, recall_score,
    confusion_matrix, matthews_corrcoef, brier_score_loss,
    roc_curve, precision_recall_curve
)
from sklearn.calibration import calibration_curve

# ------------------ Inputs ------------------
ENCODER = "models/encoder.pkl"
MODEL = "models/etv_logreg_calibrated.pkl"
THRESHOLDS = "results/thresholds.json"

EXT_FASTA = "hbvdb/pol_aa_external.fasta"   # produced by Script 5
# ------------------------------------------

# ------------------ Outputs -----------------
OUT_METRICS = "results/external_metrics_extended.tsv"
FIG_ROC = "figures/external_roc.png"
FIG_PR = "figures/external_pr.png"
FIG_CAL = "figures/external_calibration.png"
```

```
FIG_DCA = "figures/external_decision_curve.png"
# ------------------------------------------

# RT positions (true rt-numbering) extracted via RT204 anchor motif
RT_POS = [80, 84, 91, 169, 173, 180, 181, 184, 191, 194, 200, 202, 204, 215, 233, 236, 250, 256, 269]

ETV_184_SET = {"A", "G", "I", "S", "L", "F", "M", "T"}
ETV_202_SET = {"G", "I"}

RT204_REGEX = re.compile(r"Y[MVIL]DD")

def find_rt204_index(seq: str) -> int | None:
    m = RT204_REGEX.search(seq)
    if m:
        return m.start() + 1  # rt204 is the 2nd character in Y?DD
    return None

def residue_at_rt(seq: str, rt204_idx: int, rt_pos: int) -> str:
    idx = rt204_idx + (rt_pos - 204)
    if idx < 0 or idx >= len(seq):
        return "-"
    return seq[idx]

def etv_pathway_label(calls: dict[int, str]) -> int:
    lam_bg = (calls.get(180, "-") == "M") and (calls.get(204, "-") in {"V", "I"})
    etv_spec = (
        (calls.get(184, "-") in ETV_184_SET) or
        (calls.get(202, "-") in ETV_202_SET) or
        (calls.get(250, "-") == "V")
    )
    return int(lam_bg and etv_spec)

def confusion_parts(y_true, y_pred):
    tn, fp, fn, tp = confusion_matrix(y_true, y_pred).ravel()
    return tn, fp, fn, tp

def specificity(y_true, y_pred) -> float:
    tn, fp, fn, tp = confusion_parts(y_true, y_pred)
    return tn / (tn + fp) if (tn + fp) else np.nan

def ece_score(y_true, y_prob, n_bins=10) -> float:
    bins = np.linspace(0, 1, n_bins + 1)
    ece = 0.0
    for i in range(n_bins):
        lo, hi = bins[i], bins[i+1]
        mask = (y_prob >= lo) & (y_prob < hi) if i < n_bins-1 else (y_prob >= lo) & (y_prob <= hi)
        if mask.sum() == 0:
            continue
        conf = float(y_prob[mask].mean())
        acc = float(y_true[mask].mean())
        ece += (mask.sum() / len(y_prob)) * abs(acc - conf)
    return float(ece)

def compute_metrics(y_true, y_prob, thr: float) -> dict:
    y_pred = (y_prob >= thr).astype(int)
    tn, fp, fn, tp = confusion_parts(y_true, y_pred)
    return {
        "threshold": float(thr),
        "AUC_ROC": roc_auc_score(y_true, y_prob) if len(np.unique(y_true)) == 2 else np.nan,
        "AUC_PR": average_precision_score(y_true, y_prob) if len(np.unique(y_true)) == 2 else np.nan,
        "accuracy": accuracy_score(y_true, y_pred),
        "sensitivity_recall": recall_score(y_true, y_pred, zero_division=0),
        "specificity": specificity(y_true, y_pred),
```

```
        "precision": precision_score(y_true, y_pred, zero_division=0),
        "F1": f1_score(y_true, y_pred, zero_division=0),
        "MCC": matthews_corrcoef(y_true, y_pred) if len(np.unique(y_pred)) > 1 else 0.0,
        "brier": brier_score_loss(y_true, y_prob),
        "ECE_10bin": ece_score(y_true, y_prob, 10),
        "n_external": int(len(y_true)),
        "pos_external": int(y_true.sum()),
        "tn": int(tn), "fp": int(fp), "fn": int(fn), "tp": int(tp),
    }

def plot_roc(y_true, y_prob, path):
    fpr, tpr, _ = roc_curve(y_true, y_prob)
    plt.figure(figsize=(6, 5))
    plt.plot(fpr, tpr)
    plt.plot([0, 1], [0, 1], linestyle="--")
    plt.xlabel("False Positive Rate")
    plt.ylabel("True Positive Rate")
    plt.title("ROC curve (external HBVdb)")
    plt.tight_layout()
    plt.savefig(path, dpi=200)

def plot_pr(y_true, y_prob, path):
    p, r, _ = precision_recall_curve(y_true, y_prob)
    plt.figure(figsize=(6, 5))
    plt.plot(r, p)
    plt.xlabel("Recall")
    plt.ylabel("Precision")
    plt.title("Precision–Recall curve (external HBVdb)")
    plt.tight_layout()
    plt.savefig(path, dpi=200)

def plot_cal(y_true, y_prob, path):
    frac_pos, mean_pred = calibration_curve(y_true, y_prob, n_bins=10, strategy="uniform")
    plt.figure(figsize=(6, 5))
    plt.plot(mean_pred, frac_pos, marker="o")
    plt.plot([0, 1], [0, 1], linestyle="--")
    plt.xlabel("Mean predicted probability")
    plt.ylabel("Fraction of positives")
    plt.title("Calibration curve (external HBVdb)")
    plt.tight_layout()
    plt.savefig(path, dpi=200)

def decision_curve(y_true, y_prob, pts):
    n = len(y_true)
    prev = y_true.mean()
    nb_model, nb_all = [], []
    nb_none = np.zeros_like(pts, dtype=float)
    for pt in pts:
        y_pred = (y_prob >= pt).astype(int)
        tn, fp, fn, tp = confusion_parts(y_true, y_pred)
        w = pt / (1 - pt)
        nb_m = (tp / n) - (fp / n) * w
        nb_a = prev - (1 - prev) * w
        nb_model.append(nb_m)
        nb_all.append(nb_a)
    return np.array(nb_model), np.array(nb_all), nb_none

def plot_dca(y_true, y_prob, path):
    pts = np.linspace(0.01, 0.99, 99)
    nb_m, nb_a, nb_n = decision_curve(y_true, y_prob, pts)
```

```
    plt.figure(figsize=(6.5, 5))
    plt.plot(pts, nb_m, label="Model")
    plt.plot(pts, nb_a, linestyle="--", label="Treat-all")
    plt.plot(pts, nb_n, linestyle=":", label="Treat-none")
    plt.xlabel("Threshold probability")
    plt.ylabel("Net benefit")
    plt.title("Decision Curve Analysis (external HBVdb)")
    plt.legend()
    plt.tight_layout()
    plt.savefig(path, dpi=200)

def main():
    os.makedirs("results", exist_ok=True)
    os.makedirs("figures", exist_ok=True)

    if not os.path.exists(EXT_FASTA):
        raise SystemExit(f"Missing external FASTA: {EXT_FASTA} (run Script 5 first).")

    enc = joblib.load(ENCODER)
    clf = joblib.load(MODEL)

    with open(THRESHOLDS, "r") as f:
        th = json.load(f)

    ids, call_rows, y = [], [], []
    dropped_no_motif = 0

    for rec in SeqIO.parse(EXT_FASTA, "fasta"):
        sid = rec.id
        seq = str(rec.seq)

        rt204_idx = find_rt204_index(seq)
        if rt204_idx is None:
            dropped_no_motif += 1
            continue

        calls = {p: residue_at_rt(seq, rt204_idx, p) for p in RT_POS}
        ids.append(sid)
        call_rows.append([calls[p] for p in RT_POS])
        y.append(etv_pathway_label(calls))

    if not ids:
        raise SystemExit("No usable external sequences after motif filtering.")

    Xext = enc.transform(np.array(call_rows, dtype=object))
    yext = np.array(y, dtype=int)

    prob = clf.predict_proba(Xext)[:, 1]

    rows = []
    for tname in ["youdenJ", "fixed_0.5", "f1_max"]:
        thr = float(th[tname])
        m = compute_metrics(yext, prob, thr)
        row = {"dataset": "HBVdb_external", "threshold_name": tname}
        row.update({k: v for k, v in m.items() if k not in {"tn","fp","fn","tp"}})
        rows.append(row)

    df = pd.DataFrame(rows)
    df.to_csv(OUT_METRICS, sep="\t", index=False)

    print(f"[i] External usable: {len(yext)} | dropped no motif: {dropped_no_motif}")
    print(f"[i] External positives (ETV pathway): {int(yext.sum())}")
    print(f"[✓] Metrics: {OUT_METRICS}")
```

```
    if len(np.unique(yext)) == 2:
        plot_roc(yext, prob, FIG_ROC)
        plot_pr(yext, prob, FIG_PR)
        print(f"[✓] ROC: {FIG_ROC}")
        print(f"[✓] PR:  {FIG_PR}")

    plot_cal(yext, prob, FIG_CAL)
    plot_dca(yext, prob, FIG_DCA)
    print(f"[✓] Calibration: {FIG_CAL}")
    print(f"[✓] DCA: {FIG_DCA}")

if __name__ == "__main__":
    main()
```

**List of Abbreviations:**

| Abbreviation | Definition |
|---|---|
| AA | Amino acid |
| AUC | Area under the curve |
| Brier | Brier score (measure of probabilistic calibration) |
| CDS | Coding sequence |
| CI | Confidence interval |
| DCA | Decision curve analysis |
| ECE | Expected calibration error |
| ETV | Entecavir |
| FASTA | Text-based format for nucleotide or amino-acid sequences |
| F1 | Harmonic mean of precision and recall |
| GB | GenBank format |
| HBV | Hepatitis B virus |
| HBVdb | Hepatitis B Virus Database |
| MCC | Matthews correlation coefficient |
| NCBI | National Center for Biotechnology Information |
| PR | Precision–recall |
| QC | Quality control |
| ROC | Receiver operating characteristic |
| RT | Reverse transcriptase |
| SHA1 | Secure Hash Algorithm 1 (cryptographic hash function) |
| TRIPOD-AI | Transparent Reporting of a multivariable prediction model for Individual Prognosis Or Diagnosis – Artificial Intelligence extension |
| YMDD | Tyrosine–methionine–aspartate–aspartate motif |
